# RNA editing enzyme ADAR1 controls miR-381-3p-mediated expression of multidrug resistance protein MRP4 *via* regulation of circRNA in human renal cells

**DOI:** 10.1016/j.jbc.2022.102184

**Published:** 2022-06-23

**Authors:** Yuji Omata, Maseri Okawa, Mai Haraguchi, Akito Tsuruta, Naoya Matsunaga, Satoru Koyanagi, Shigehiro Ohdo

**Affiliations:** 1 Department of Pharmaceutics, Faculty of Pharmaceutical Sciences, Kyushu University, Fukuoka, Japan; 2 Department of Glocal Healthcare Science, Faculty of Pharmaceutical Sciences, Kyushu University, Fukuoka, Japan; 3 Department of Clinical Pharmacokinetics, Faculty of Pharmaceutical Sciences, Kyushu University, Fukuoka, Japan

**Keywords:** RNA editing, microRNA, ABC transporter, posttranscriptional regulation, RNA splicing, circular RNA, A-to-I, adenosine-to-inosine, circRNA, circular RNA, dsRBD, dsRNA-binding domain, MRP4, multidrug resistance–associated protein 4, OX, overexpressing, RBP, RNA-binding protein, RIP, RNA immunoprecipitation, RPTEC, renal proximal tubular epithelial cell

## Abstract

Multidrug resistance–associated protein 4 (MRP4), a member of the C subfamily of ATP-binding cassette transporters, is highly expressed in the kidneys of mammals and is responsible for renal elimination of numerous drugs. Adenosine deaminase acting on RNA 1 (ADAR1) has been reported to regulate gene expression by catalyzing adenosine-to-inosine RNA editing reactions; however, potential roles of ADAR1 in the regulation of MRP4 expression have not been investigated. In this study, we found that downregulation of ADAR1 increased the expression of MRP4 in human renal cells at the posttranscriptional level. Luciferase reporter assays and microarray analysis revealed that downregulation of ADAR1 reduced the levels of microRNA miR-381-3p, which led to the corresponding upregulation of MPR4 expression. Circular RNAs (circRNAs) are a type of closed-loop endogenous noncoding RNAs that play an essential role in gene expression by acting as miRNA sponges. We demonstrate that ADAR1 repressed the biogenesis of circRNA circHIPK3 through its adenosine-to-inosine RNA editing activity, which altered the secondary structure of the precursor of circHIPK3. Furthermore, *in silico* analysis suggested that circHIPK3 acts as a sponge of miR-381-3p. Indeed, we found overexpression of circHIPK3 induced the expression of MRP4 through its interference with miR-381-3p. Taken together, our study provides novel insights into regulation of the expression of xenobiotic transporters through circRNA expression by the RNA editing enzyme ADAR1.

Multidrug resistance–associated protein 4 (MRP4), encoded by the *ABCC4* gene, is one of the ABC transporters and highly expressed in proximal renal tubules, bone marrow, and the brain of mammals (1, 2, 3). MRP4 recognizes a variety of compounds as substrates and transports them to the extracellular fluids (4). In the kidney, MRP4 participates in the elimination of many drugs, affecting their renal clearance. The expression of MRP4 is regulated in a tissue-dependent manner. Highly expressed transcription factors, such as PPARα, NRF2, and AHR, are thought to be involved in the transcription of *ABCC4* mRNA in human renal cells (5). In addition to transcriptional regulation, posttranscriptional regulation has been demonstrated to be involved in MRP4 expression (6).

MicroRNA (miRNA) is a small noncoding RNA that binds to the 3′ untranslated region (3′UTR) of target mRNA, inducing mRNA degradation or transcriptional inhibition (7). Recent studies suggest that some members of the ABC transporter family are subject to miRNA-mediated gene regulation (8). For example, the downregulation of miR-298 in doxorubicin-resistant human breast cancer cells induced high expression of P-glycoprotein, conferring doxorubicin resistance (9). Several miRNAs are also found to regulate the expression of MRP4 (8). miR-124a and miR-506 reduce MRP4 protein levels in HEK293T/17 cells (10), suggesting the role of miRNAs in regulating the expression of xenobiotic transporters in the human kidney. Although there are many reports demonstrating miRNA-mediated posttranscriptional regulation of ABC transporters, the regulatory mechanisms underlying the expression and function of miRNA have yet to be elucidated.

Circular RNA (circRNA) is a single-stranded noncoding RNA that forms a covalently closed loop structure having neither 5′ to 3′ polarity nor a polyadenylated tail (11). Numerous circRNAs act as a “sponge” of miRNAs, becoming a novel candidate for regulating the function of miRNAs (12, 13, 14). The biogenesis of circRNAs is different from canonical splicing of linear RNAs; circRNAs are formed by back-splicing, in which a downstream 5′ splice site is joined to an upstream 3′ splice site (15). Back-splicing requires the canonical 5′ and 3′ splice sites (16) and the canonical spliceosome assembly (17). In general, the efficiency of back-splicing is much lower than that of canonical splicing (18); however, due to their high stability, some circRNAs exhibit higher expression than their counterpart linear mRNAs (14). RNA pairing at inverted repetitive elements, such as *Alu* elements in primates, in introns flanking the circularized exon is hypothesized to promote circRNA biogenesis because it brings the back-splice sites into close proximity (15, 19).

In addition to these *cis*-regulatory elements, some RNA-binding proteins (RBPs) act as *trans*-acting factors of back-splicing. Numerous circRNAs are upregulated by quaking (QKI) during epithelial-mesenchymal transformation (20). QKI binds to the flanking intron of circRNA-forming exons and then forms a dimer, bringing the back-splice sites closer together. Another example of circRNA-related RBPs is adenosine deaminase acting on RNA 1 (ADAR1), which suppresses the biogenesis of several circRNAs (21, 22). ADAR1 catalyzes adenosine-to-inosine (A-to-I) RNA editing, which is the most prevalent nucleotide conversion in mammals (23). Inosine behaves like guanosine as it forms a base pair with cytidine. Thus, A-to-I RNA editing leads to a codon change, alternative splicing, and alteration of RNA secondary structure (24). A-to-I RNA editing on the double-stranded RNA formed in the precursor of circRNAs diminishes the complementarity and disrupts RNA pairing, leading to the decreased biogenesis of circRNAs (21, 22). Although it has broad substrate-recognition capacity and ubiquitous expression in multiple tissues (23, 25, 26), only a few studies reported the involvement of ADAR1 in circRNA biogenesis (27, 28, 29).

Our previous study demonstrated that ADAR1 regulated the expression of P-glycoprotein in human renal proximal tubular epithelial cells (RPTECs) (30). During the analysis of the role of ADAR1 in the regulation of renal expression of xenobiotic transporters, we also found that the expression of MRP4 protein was increased by the downregulation of ADAR1. ADAR1 controlled MRP4 expression at the posttranscriptional level. Therefore, we investigated the underlying mechanism of ADAR1-mediated MRP4 expression in RPTECs by focusing on the sponging function of circRNA against miRNA.

## Results

### Downregulation of ADAR1 increases the expression of MRP4 through the 3′UTR of ABCC4 mRNA in human RPTECs

**Figure 1:**
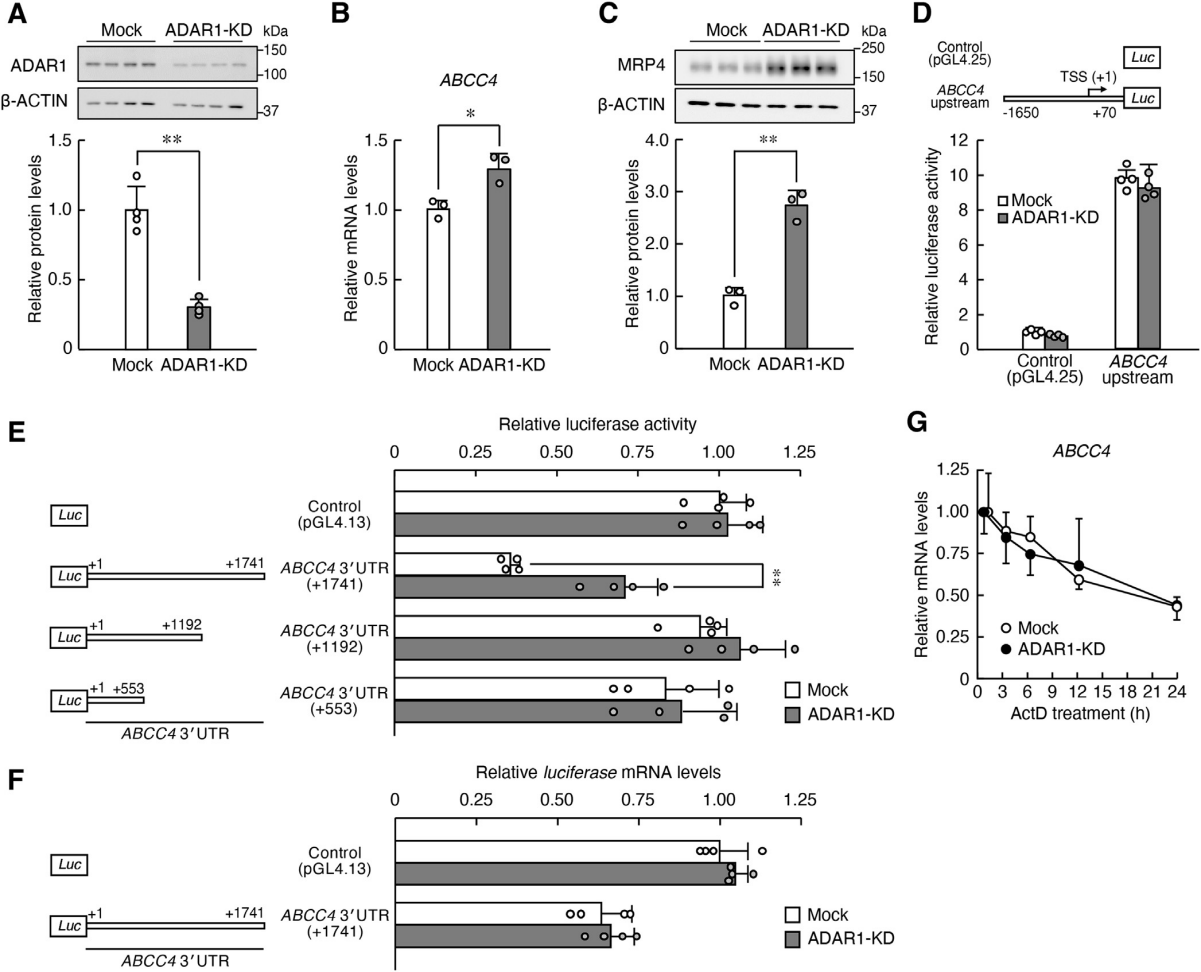
**ADAR1 regulates the expression of MRP4 through the 3′UTR of *ABCC4* mRNA in RPTECs.***A*, construction of ADAR1-knockdown (KD) RPTECs with stable expression of shRNA. The expression level of ADAR1 protein was normalized to that of β-ACTIN. Values are the mean with SD (n = 4). The value of ADAR1 in mock-transduced RPTECs was set at 1.0. ∗∗*p* < 0.01; significant difference between the two groups (*t*_6_ = 7.694, *p* < 0.001; unpaired *t* test, two sided). *B*, the mRNA levels of *ABCC4* in mock-transduced and ADAR1-KD RPTECs. The mRNA levels were normalized to those of 18S rRNA. Values are the mean with SD (n = 3). ∗*p* < 0.05; significant difference between the two groups (*t*_4_ = 4.178, *p* = 0.014; unpaired *t* test, two sided). *C*, the protein levels of MRP4 in mock-transduced and ADAR1-KD RPTECs. The protein levels were normalized to those of β-ACTIN. Values are the mean with SD (n = 3). ∗∗*p* < 0.01; significant difference between the two groups (*t*_4_ = 9.165, *p* < 0.001; unpaired *t* test, two sided). *D*, the luciferase activity of *ABCC4* upstream::Luc in mock-transduced and ADAR1-KD RPTECs. Schematic diagrams of luciferase reporter constructs containing the 5′ flanking region of the *ABCC4* gene are shown in the *upper panel*. The numbers in the *upper panel* indicate the distance in base pairs from the putative transcription start site (TSS, +1). Values are the mean with SD (n = 4). *E*, the luciferase activity of *ABCC4* 3′UTR::Luc in mock-transduced and ADAR1-KD RPTECs. Schematic diagrams of luciferase reporter constructs containing varying length of the 3′UTR of the *ABCC4* gene are shown in the *left panel*. The *numbers* in the *left panel* indicate the distance in base pairs from the stop codon (+1). Values are the mean with SD (n = 4). ∗∗*p* < 0.01; significant difference between the indicated groups (*F*_7, 24_ = 14.807, *p* < 0.001; ANOVA with Tukey–Kramer’s post hoc test). *F*, the mRNA levels of the *Luciferase* gene in mock-transduced and ADAR1-KD RPTECs transfected with luciferase reporter constructs containing the full-length *ABCC4* 3′UTR. The mRNA levels of the *luciferase* gene were normalized to those of *Renilla Luciferase*. Values are the mean with SD (n = 4). *G*, the stability of *ABCC4* mRNA in mock-transduced and ADAR1-KD RPTECs. Cells were treated with 5 μM actinomycin D (ActD) and RNA was extracted at the indicated time points. The mRNA levels were normalized to those of 18S rRNA. Values are the mean with SD (n = 3, 4).

In our previous study (30), ADAR1-knockdown (KD) RPTECs were prepared by stable expression of small hairpin RNA (shRNA). No notable bands derived from ADAR1-p150 were detected in RPTECs, but the expression levels of the ADAR1-p110 isoform were significantly reduced to 40% in the anti-ADAR1 shRNA-transduced RPTECs (*p* < 0.01, Fig. 1*A*). Therefore, we used these cells to investigate the role of ADAR1 in the regulation of MRP4 expression. In the ADAR1-KD RPTECs, the mRNA levels of *ABCC4* significantly increased in comparison with mock-transduced RPTECs (*p* < 0.05, Fig. 1*B*). The expression levels of MRP4 increased approximately 2.7-fold in ADAR1-KD RPTECs (*p* < 0.01, Fig. 1*C*).

The protein level of MRP4 is regulated at nearly all stages of the gene expression process (5). To investigate whether ADAR1 regulates the expression of MRP4 at the transcriptional level, we constructed a luciferase reporter vector containing the 5′ flanking region of the human *ABCC4* gene (*ABCC4* upstream::Luc) spanning from bp −1650 to +70 (the number is the distance in base pairs from the putative transcription start site, +1). The 5′ flanking region contains several response elements for NRF2, PPARα, and AHR (Fig. S1*A*), which are known to regulate transcription of the human *ABCC4* gene (5). The expression levels for these transcriptional factors were not significantly different between mock-transduced and ADAR1-KD RPTECs (Fig. S1*B*). Consistent with this result, luciferase activity derived from *ABCC4* upstream::Luc was not significantly altered in ADAR1-KD RPTECs (Fig. 1*D*). This suggests that ADAR1 is involved in the posttranscriptional process of MRP4 expression rather than transcriptional regulation of the *ABCC4* gene. Therefore, we changed our focus to the posttranscriptional regulation mechanism promoting MRP4 expression in ADAR1-KD cells.

As translational efficiency is often regulated *via* 3′UTR of mRNA containing target sites of miRNAs (7), we explored whether ADAR1 acts on the 3′UTR of *ABCC4* gene. To achieve this, the varying lengths of the 3′UTR of *ABCC4* gene from bp +1 to +1741 (*ABCC4* 3′UTR +1741::Luc), from +1 to +1192 (*ABCC4* 3′UTR +1192::Luc), and from +1 to +553 (*ABCC4* 3′UTR +553::Luc) were cloned downstream of the *luciferase* gene into the pGL4.13 reporter vector (the nucleotide immediately after the stop codon in exon 30 of the human *ABCC4* gene was defined as +1). The luciferase activity of *ABCC4* 3′UTR +1741::Luc significantly decreased in comparison with the control pGL4.13 reporter vector without containing 3′UTR (Fig. 1*E*), indicating that the 3′UTR of *ABCC4* mRNA represses the translation of MRP4 protein. The reporter activity derived from *ABCC4* 3′UTR +1741::Luc in ADAR1-KD RPTECs was significantly higher than that in mock-transduced cells (*p* < 0.01, Fig. 1*E*), but downregulation of ADAR1 had negligible effects on the reporter activities of *ABCC4* 3′UTR +1192::Luc and *ABCC4* 3′UTR +553::Luc. This suggests that the 3′UTR of *ABCC4* mRNA from +1193 to +1741 is responsible for the ADAR1-mediated regulation of MRP4 expression. The levels of *Luciferase* mRNA derived from *ABCC4* 3′UTR +1741::Luc were not significantly different between mock-transduced and ADAR1-KD RPTECs (Fig. 1*F*). Furthermore, the stability of *ABCC4* mRNA was also not significantly affected by the downregulation of ADAR1 (Fig. 1*G*). Thus, the increased luciferase activity derived from *ABCC4* 3′UTR +1741 reporter vectors in ADAR1-KD cells was due to increased translational efficiency rather than the stabilization of mRNA.

### miR-381-3p is involved in the ADAR1-mediated translational regulation of ABCC4 gene

**Figure 2:**
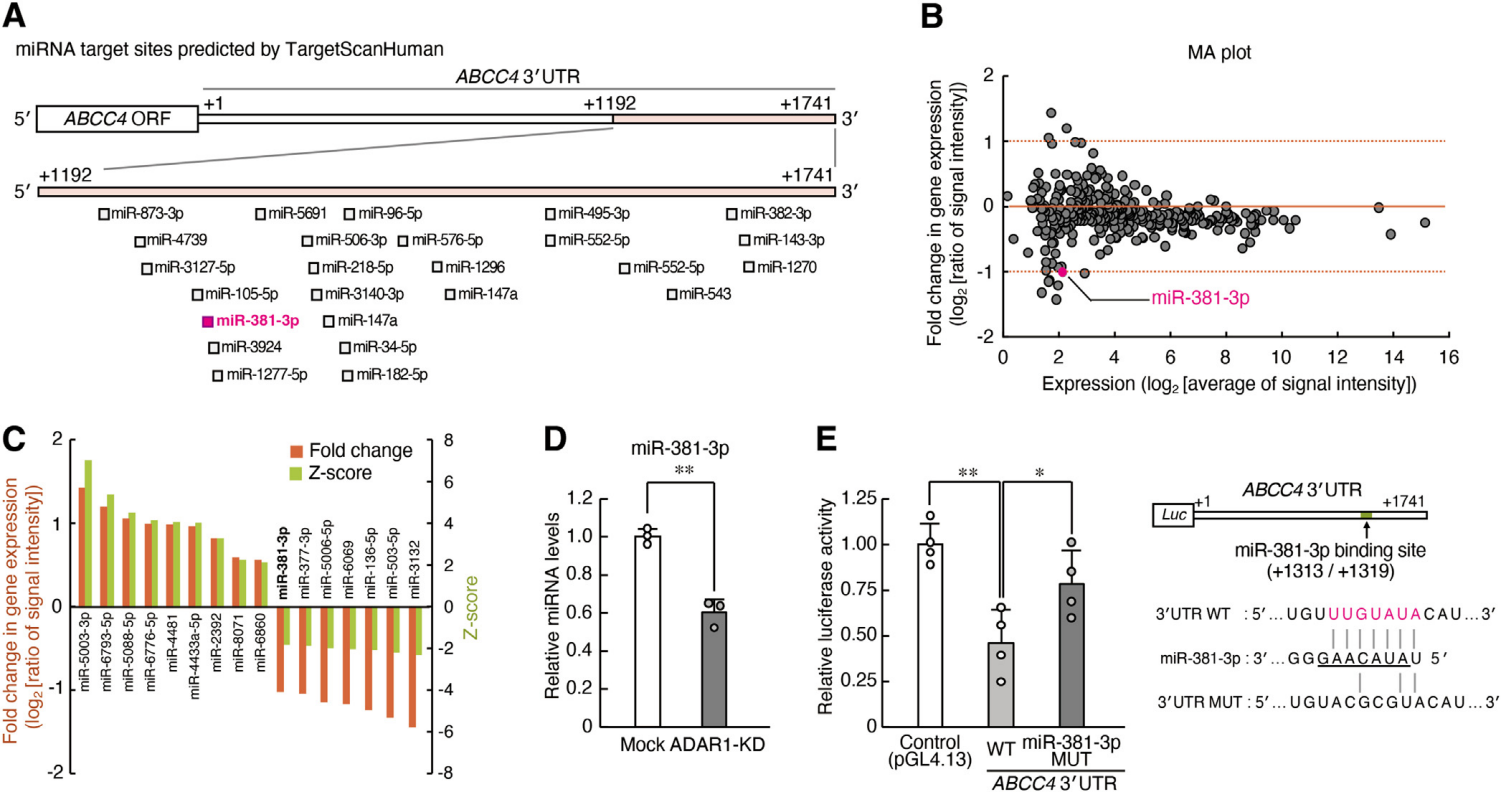
**ADAR1 regulates the translation of *ABCC4* mRNA into MRP4 protein through mediating miR-381-3p expression.***A*, schematic diagram showing the putative target sites of miRNAs in *ABCC4* 3′UTR from +1192 to +1741. The *numbers* indicate the distance in base pairs from the stop codon (+1). The miRNA target sites were predicted with TargetScanHuman (Release 7.2). *B*, MA plot of all miRNAs expressed in mock-transduced and ADAR1-KD RPTECs. Data were obtained from microarray analysis (accession number GSE192692). The criteria for upregulated miRNAs were set at a ratio of 2-fold or Z-score of 2.0; the criteria for downregulated miRNAs were set at a ratio of 0.5-fold or Z-score of −2.0. *C*, differentially expressed miRNAs between mock-transduced and ADAR1-KD RPTECs in the microarray analysis. *D*, the expression levels of miR-381-3p in mock-transduced and ADAR1-KD RPTECs. The expression levels were assessed by qRT-PCR and normalized to those of miR-191-5p, which was used as an endogenous control of miRNA and normalizer of RT and PCR steps (32). Values are the mean with SD (n = 3). ∗∗*p* < 0.01; significant difference between the two groups (*t*_4_ = 8.567, *p* = 0.001; unpaired *t* test, two sided). *E*, the luciferase activity in RPTECs transfected with wildtype (WT) or mutant (MUT) *ABCC4* 3′UTR::Luc. Schematic diagrams of luciferase reporter constructs containing WT or MUT (mutation in miR-381-3p target site) 3′UTR of the *ABCC4* gene are shown in the *right panel*. The *numbers* in the *right panel* indicate the distance in base pairs from the stop codon (+1). Values are the mean with SD (n = 4). ∗∗*p* < 0.01, ∗*p* < 0.05; significant difference between the indicated groups (*F*_*2*, 9_ = 11.252, *p* = 0.004; ANOVA with Tukey–Kramer’s post hoc test).

Direct sequencing analysis revealed no ADAR1-mediated A-to-I RNA editing sites in the 3′UTR of *ABCC4* mRNA (Fig. S2). Therefore, we investigated the possibility that ADAR1 regulates the expression of MRP4 through the mediation of miRNAs. The computational analysis using TargetScanHuman (31) identified 25 miRNAs whose target sites are located in the *ABCC4* mRNA 3′UTR from bp +1193 to +1741 (Fig. 2*A*). Microarray analysis also showed 16 miRNAs that were differentially expressed between mock-transduced and ADAR1-KD RPTECs. For this analysis, two criteria were set for selecting upregulated miRNAs: Z-score of 2.0 or higher and ratio of 2-fold or greater; and for downregulated miRNAs: Z-score of −2.0 or lower and ratio of 0.5 or lower. The expression levels of nine miRNAs increased in ADAR1-KD RPTECs, whereas those of seven miRNAs decreased in ADAR1-KD RPTECs (Fig. 2, *B* and *C*). By combining these results, we focused on miR-381-3p as a possible candidate to regulate the translation of *ABCC4* gene. The expression of miR-381-3p in ADAR1-KD RPTECs was significantly lower than that in mock-transduced RPTECs (*p* < 0.01, Fig. 2*D*). In this analysis, the expression levels of miR-381-3p were normalized to those of miR-191-5p, which is recommended as an endogenous control of miRNA and normalizer of RT and PCR steps (32). In addition, the luciferase activity of *ABCC4* 3′UTR +1741::Luc in RPTECs was significantly increased by mutation of the target site of miR-381-3p (*p* < 0.05, Fig. 2*E*). Therefore, the translation of the *ABCC4* gene in RPTECs is repressed by miR-381-3p whose expression is under the control of ADAR1. This RNA editing enzyme may elevate the expression of miR-381-3p.

### circHIPK3 mediates the ADAR1-induced regulation of miR-381-3p levels

**Figure 3:**
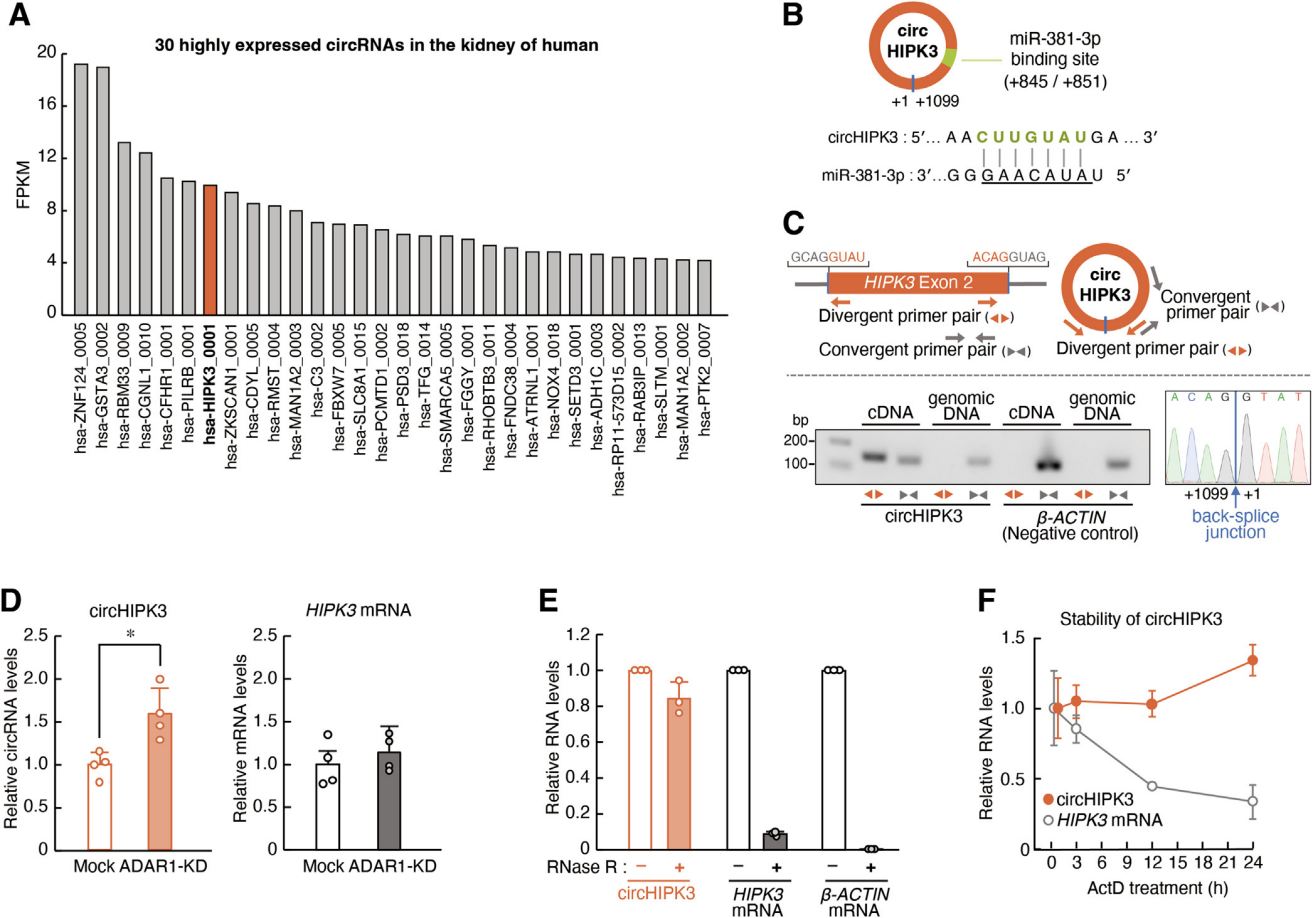
**ADAR1 regulates the expression of circHIPK3 in RPTECs.***A*, 30 highly expressed circRNAs in the human kidney registered in circAtlas database. FPKM: fragments per kilobase of exon per million mapped reads. *B*, schematic diagram showing the putative target site of miR-381-3p in circHIPK3. The interaction of miR-381-3p and the circRNAs was predicted using starBase v2.0. *C*, validation of circHIPK3 detection by RT-PCR. Divergent primers amplified the circHIPK3 back-splice junction sequence in the cDNA but not the genomic DNA. Convergent primers amplified both mRNA in cDNA and genomic DNA. Sanger sequencing confirmed the back-splice junction sequence of the PCR products with the divergent primers (*right panel*). *D*, the expression levels of circHIPK3 and *HIPK3* mRNA in mock-transduced and ADAR1-KD RPTECs. The expression levels were normalized to those of 18S rRNA. Values are the mean with SD (n = 4). ∗*p* < 0.05; significant difference between the two groups (*t*_*6*_ = 3.494, *p* = 0.013 for circHIPK3; unpaired *t* test, two sided). *E*, qRT-PCR analysis for the abundance of circHIPK3, *HIPK3* mRNA, and *β-ACTIN* mRNA in RPTECs treated with RNase R. The amounts of target RNAs were normalized to those in the mock treatment. *F*, the stability of circHIPK3 and *HIPK3* mRNA in RPTECs. Cells were treated with 5 μM actinomycin D (ActD), and RNA was extracted at the indicated time points. The expression levels were normalized to those of 18S rRNA. Values are the mean with SD (n = 4).

To investigate the underlying mechanism of ADAR1-induced regulation of miR-381-3p in RPTECs, we focused on circRNAs for the following reasons: ADAR1 regulates the biogenesis of circRNAs by RNA editing (21, 22); circRNAs function as a “sponge” of miRNAs and interfere with their binding to target RNAs (12, 13); and some circRNAs prevent the function and expression of miR-381-3p (33, 34, 35). As circRNAs are expressed in tissue- and cell type–dependent manners (36), we investigated the expression profile of circRNAs in the kidney of human using the circRNA expression database circAtlas (37). In the database, 30 circRNAs were registered as exhibiting higher expression in the human kidney (Fig. 3*A*). Among them, circHIPK3 (circRNA ID: hsa_circ_0000284) was identified by starBase v2.0 (38) as having a target site for miR-381-3p (Fig. 3*B*). Therefore, we investigated whether the expression of circHIPK3 was altered in ADAR1-KD RPTECs. For quantification of circHIPK3 levels, we designed circHIPK3-specific divergent primers to span the circHIPK3 back-splice junction (Fig. 3*C* *upper*), which is a general method to specifically amplify circRNAs without amplification of the counterpart linear mRNA (39). The expression of circHIPK3 in RPTECs was detected by RT-PCR with the divergent primers (Fig. 3*C* *left*), and the circHIPK3-specific sequence at the back-splice junction was also confirmed by Sanger sequencing of the PCR product amplified with the divergent primers (Fig. 3*C* *right*). The levels of circHIPK3 significantly increased in ADAR1-KD RPTECs (*p* < 0.05, Fig. 3*D*), whereas the expression of the counterpart linear *HIPK3* mRNA was not significantly different between mock-transduced and ADAR1-KD RPTECs (Fig. 3*D*). To investigate whether the expression of circHIPK3 is specifically increased in ADAR1-KD RPTECs, we also assessed the expression levels of other circRNAs by designing divergent primers. Of the 30 circRNAs listed in Figure 3*A*, 22 were detected in RPTECs, but there were no circRNAs whose expression were elevated by downregulation of ADAR1 (Fig. S3*A*). Therefore, among the circRNAs expressed in RPTECs, circHIPK3 may be more specifically upregulated in ADAR1-KD RPTECs. In contrast to linear mRNAs such as *HIPK3* and *β-ACTIN*, circHIPK3 and other circRNAs were resistant to digestion by RNase R, a 3′ to 5′ exonuclease (Figs. 3*E* and S3*B*) because circRNAs do not have 5′ or 3′ ends. High stability of circHIPK3 was also confirmed in RPTECs after treatment with 5 μM actinomycin D, a transcription inhibitor (Fig. 3*F*).

**Figure 4:**
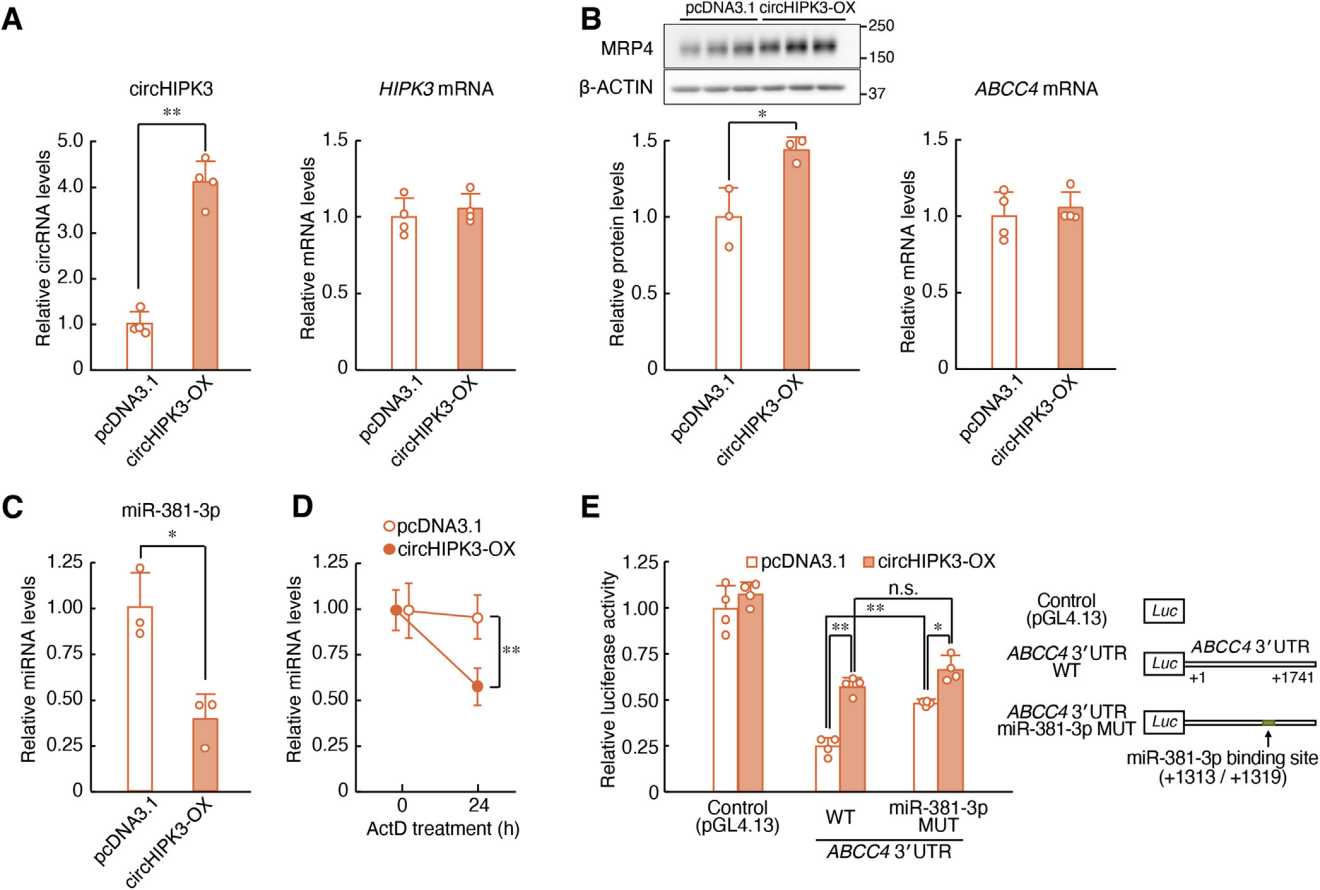
**circHIPK3 regulates the expression of MRP4 protein *via* miR-381-3p.***A*, the expression levels of circHIPK3 and *HIPK3* mRNA in RPTECs transfected with circHIPK3-expression vector or empty vector (pcDNA3.1). The expression levels were normalized to those of 18S rRNA. Values are the mean with SD (n = 4). ∗∗*p* < 0.01; significant difference between the two groups (*t*_6_ = 11.286, *p* < 0.001 for circHIPK3; unpaired *t* test, two sided). *B*, the protein levels of MRP4 and mRNA levels of *ABCC4* in pcDNA3.1-transfected and circHIPK3-overexpressing (OX) RPTECs. The protein levels were normalized to those of β-ACTIN, and the mRNA levels were normalized to those of 18S rRNA. Values are the mean with SD (n = 3, 4). ∗*p* < 0.05; significant difference between the two groups (*t*_4_ = 3.697, *p* = 0.021 for MRP4; unpaired *t* test, two sided). *C*, the expression levels of miR-381-3p in pcDNA3.1 (control)-transfected and circHIPK3-OX RPTECs. The expression levels were normalized to those of miR-191-5p. Values are the mean with SD (n = 3). ∗*p* < 0.05; significant difference between the two groups (*t*_4_ = 4.473, *p* = 0.011; unpaired *t* test, two sided). *D*, the stability of miR-381-3p in pcDNA3.1-transfected or circHIPK3-OX RPTECs. The transfected cells were treated with 5 μM actinomycin D (ActD) and RNA was extracted at the indicated time points. The expression levels were normalized to those of miR-191-5p. The value at 0 h (the time of initiation of ActD treatment) was set at 1.0. Values are the mean with SD (n = 4). ∗∗*p* < 0.01; significant difference between the indicated groups (*F*_3, 12_ = 11.433, *p* < 0.001; ANOVA with Tukey–Kramer’s post hoc test). *E*, the luciferase activity derived from *ABCC4* 3′UTR WT::Luc and *ABCC4* 3′UTR MUT::Luc in pcDNA3.1-transfected and circHIPK3-OX RPTECs. Schematic diagrams of luciferase reporter constructs containing the 3′UTR of *ABCC4* gene are shown in the *right panel*. The *numbers* in the *right panel* indicate the distance in base pairs from the stop codon (+1). Values are the mean with SD (n = 4). ∗∗*p* < 0.01, ∗*p* < 0.05; significant difference between the indicated groups (*F*_5, 18_ = 83.630, *p* < 0.001; ANOVA with Tukey–Kramer’s post hoc test). n.s., not significant.

Next, we investigated whether circHIPK3 is involved in the regulation of MRP4 expression. For this experiment, we constructed circHIPK3 expression vectors in which exon 2 of the *HIPK3* gene and its flanking introns containing inverted complementary *Alu* elements were inserted into the multicloning site of pcDNA3.1. The transcripts derived from the expression vectors were considered to be back-spliced and form circHIPK3. The expression of circHIPK3 significantly increased in the RPTECs transfected with the circHIPK3 expression vectors (*p* < 0.01, Fig. 4*A*). The expression of MRP4 also significantly increased in the circHIPK3-overexpressing (OX) RPTECs (*p* < 0.05, Fig. 4*B* *left*), whereas *ABCC4* mRNA levels were not affected by circHIPK3 expression (Fig. 4*B* *right*). Transfection of RPTECs with circHIPK3 expression vectors also reduced the levels of miR-381-3p (*p* < 0.05, Fig. 4*C*). The stability of miR-381-3p was also decreased in circHIPK3-OX RPTECs (*p* < 0.01, Fig. 4*D*), suggesting that circHIPK3 decreases the expression levels of miR-381-3p resulting from its destabilization.

A significant increase in the luciferase activity of *ABCC4* 3′UTR::Luc was detected in RPTECs when cells were transfected with circHIPK3 expression vectors (*p* < 0.01, Fig. 4*E*). The luciferase activity of *ABCC4* 3′UTR::Luc was also increased by mutation of the target site of miR-381-3p (Fig. 4*E*). The circHIPK3-induced increase in luciferase activity of *ABCC4* 3′UTR::Luc was comparable with that derived from miR-381-3p target site–mutated *ABCC4* 3′UTR::Luc in circHIPK3-OX RPTECs. This suggests that circHIPK3 exerts its sponging function against miR-381-3p, thereby preventing the miR-381-3p-mediated repression of translation from *ABCC4* mRNA to MRP4 protein. This circHIPK3/miR-381-3p axis may play an essential role in the ADAR1-mediated regulation of MRP4 expression in human renal cells.

### ADAR1 regulates the expression of MRP4 with its RNA editing and dsRNA binding ability

**Figure 5:**
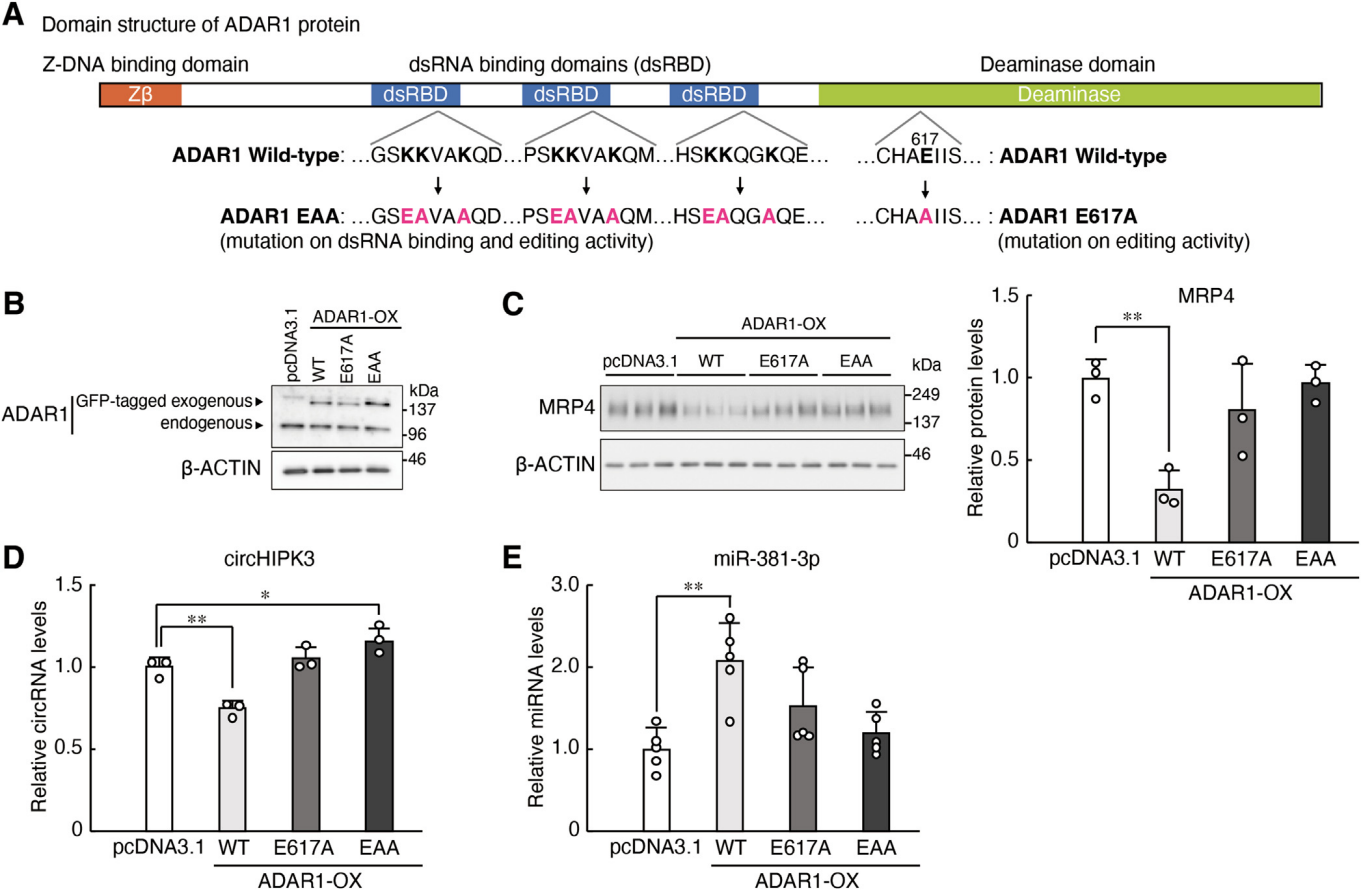
**ADAR1 regulates the expression of MRP4 with its editing and dsRNA binding ability.***A*, schematic diagram of the domain structure of human ADAR1. ADAR1 has a Z-DNA binding domain, three repeats of double-stranded RNA (dsRNA)-binding domain (dsRBD), and a catalytic deaminase domain. The E617A point mutation abolishes A-to-I RNA editing activity (E617A). Mutation of KKxxK motif in dsRBD to EAxxA abolishes dsRNA binding ability of ADAR1, thus deficient in A-to-I RNA editing activity. *B*, construction of ADAR1-overexpression (OX) RPTECs. pcDNA3.1, wildtype (WT), E617A mutant, or EAA mutant GFP-tagged ADAR1-expressing vectors were transfected into RPTECs with electroporation. Bands derived from endogenous ADAR1 and GFP-tagged exogenous ADAR1 (WT, E617A or EAA) were detected in the Western blotting image. The protein levels of β-ACTIN are shown as loading control. *C*, the protein levels of MRP4 in pcDNA3.1-transfected and ADAR1 (WT, E617A, EAA)-OX RPTECs. The protein levels were normalized to those of β-ACTIN. Values are the mean with SD (n = 3). ∗∗*p* < 0.01; significant difference between the indicated groups (*F*_3, 8_ = 9.456, *p* = 0.005; ANOVA with Dunnett’s post hoc test). *D*, the expression levels of circHIPK3 in pcDNA3.1-transfected and ADAR1 (WT, E617A, EAA)-OX RPTECs. The expression levels were normalized to those of 18S rRNA. Values are the mean with SD (n = 3). ∗∗*p* < 0.01, ∗*p* < 0.05; significant difference between the indicated groups (*F*_3, 8_ = 25.460, *p* < 0.001; ANOVA with Dunnett’s post hoc test). *E*, the expression levels of miR-381-3p in pcDNA3.1-transfected and ADAR1 (WT, E617A, EAA)-OX RPTECs. The expression levels were normalized to those of miR-191-5p. Values are the mean with SD (n = 5). ∗∗*p* < 0.01; significant difference between the indicated groups (*F*_3, 16_ = 7.647, *p* = 0.002; ANOVA with Dunnett’s post hoc test).

ADAR1-mediated gene expression is known to be not only in editing-dependent manner but also in editing-independent manner; ADAR1 enables to modulate gene expression only through the binding to double-stranded RNA (dsRNA) (40, 41). To investigate how ADAR1 acts on RNAs and modulate their expressions, we constructed ADAR1 expression vectors in which RNA editing activity or dsRNA binding ability was deficient. In the structure of ADAR1 protein, a deaminase domain and three repeats of dsRNA-binding domain (dsRBD) are crucial for RNA editing (Fig. 5*A*). A point mutation of Glu617 to Ala617 (E617A) is sufficient to inactivate the RNA editing activity (42, 43). Mutations of KKxxK motif in each dsRBD to EAxxA motif (EAA mutation) abolish the dsRNA binding ability, thus leading to deficiency of RNA editing activity (44). In contrast to ADAR1-KD cells (Fig. 1*C*), the expression of MRP4 was significantly decreased in wildtype ADAR1-OX RPTECs (*p* < 0.01, Fig. 5, *B* and *C*), but the expression levels of MRP4 were not significantly decreased in E617A-mutated ADAR1-OX and EAA-mutated ADAR1-OX RPTECs as compared with those in wildtype ADAR1-OX cells (Fig. 5*C*). Similar negligible effects of E617A-mutated and EAA-mutated ADAR1 were observed on the expressions of circHIPK3 (Fig. 5*D*) and miR-381-3p (Fig. 5*E*). These data suggested that both RNA editing activity and dsRNA binding ability of ADAR1 are indispensable for regulation of MRP4 expression *via* the circHIPK3/miR-381-3p pathway.

### ADAR1 suppresses circHIPK3 formation by inhibiting base pairing in its precursor RNA

**Figure 6:**
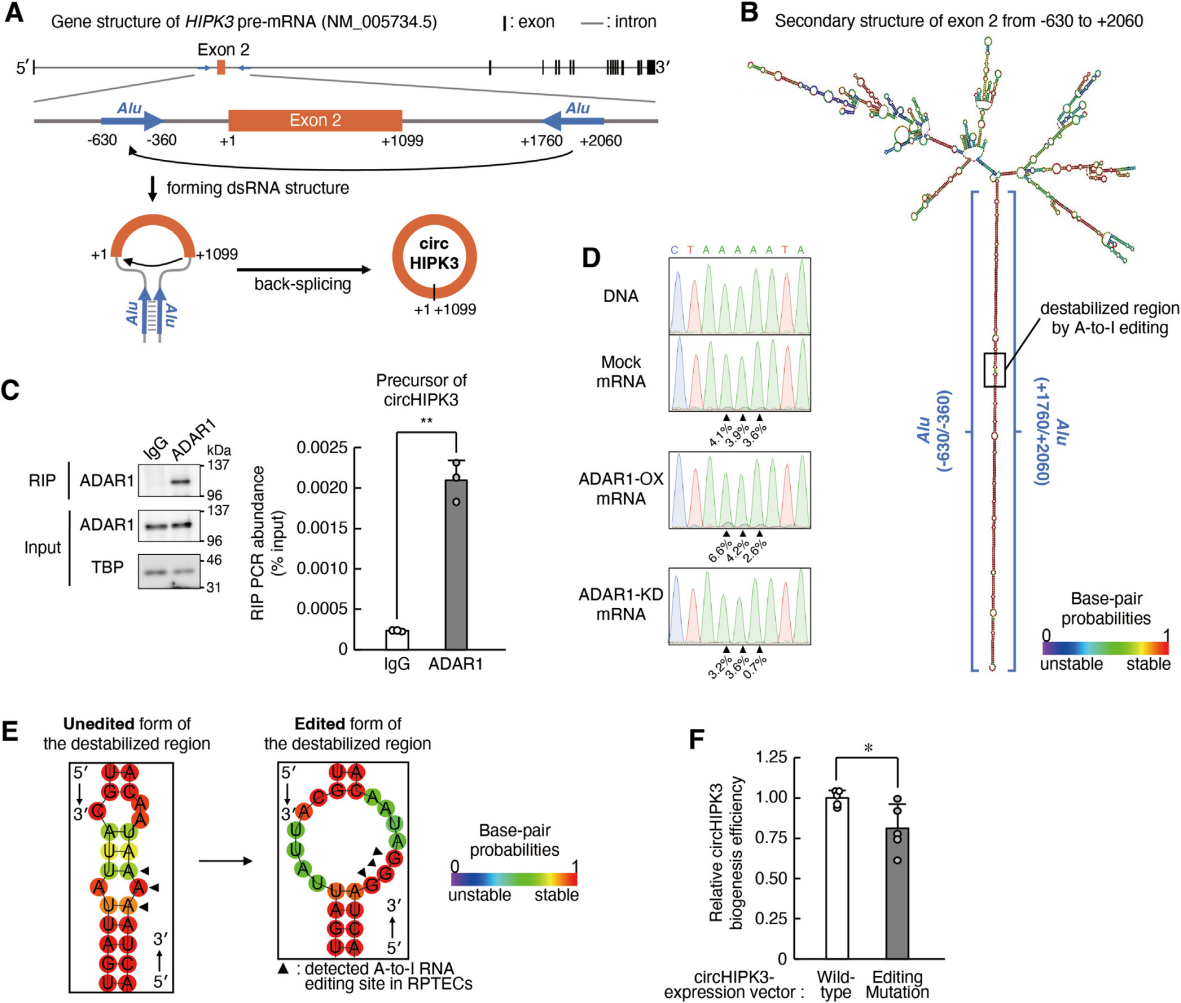
**ADAR1 suppresses circHIPK3 formation by inhibiting base pairing in its precursor RNA.***A*, schematic diagram of the genomic region of human *HIPK3* exon 2 and its flanking introns with inverted *Alu* elements (*upper panel*). CircHIPK3 is formed by back-splicing, in which a 5′ splice site in the downstream region of exon 2 joins to the 3′ splice site in the upstream region of exon 2 (*lower panel*). *B*, *in silico* analysis for prediction of RNA secondary structure of the *HIPK3* pre-mRNA (the region from −630 to +2060 of *HIPK3* exon 2) by RNAfold. The minimum free energy structure with base-pair probabilities was calculated to have the lowest value of free energy. Base-pair probabilities are shown by a color spectrum. *C*, ADAR1 binds to the precursor of circHIPK3. RPTECs were infected with lentiviral particles expressing precursor of circHIPK3 and RIP was conducted. *Left panel* shows Western blotting analysis of ADAR1-RIP immunoprecipitates. *Right panel* shows qRT-PCR analysis of ADAR1-RIP immunoprecipitates using pre-circHIPK3 targeting primers listed in Table 1. Values are the mean with SD (n = 3). ∗∗*p* < 0.01; significant difference between the two groups (*t*_4_ = 13.102, *p* < 0.001; unpaired *t* test, two sided). *D*, electropherograms from direct sequencing of the PCR-amplified product of precursor of circHIPK3 derived from lentivirus. The sequence data were obtained from mock-transduced, ADAR1-overexpression (OX), and ADAR1-KD RPTECs. Triangles indicate the ADAR1-mediated A-to-I RNA editing sites detected in RPTECs. Percentage represents the editing frequency calculated by the peak height of “G” peak over sum of “A” and “G” peak heights. Electropherograms were aligned using SnapGene Viewer. *E*, *in silico* prediction of RNA secondary structure around the editing sites in precursor of circHIPK3. *Left* and *right panels* show the unedited and edited forms, respectively. The minimum free energy structure with base-pair probabilities was calculated to have the lowest value of free energy. Base-pair probabilities are shown by a color spectrum. *F*, the biogenesis efficiency of circHIPK3 derived from circHIPK3-expression vector with and without the mutation in *Alu* elements. The efficiency was calculated as the ratio of the expression levels of circHIPK3 to that of precursor of circHIPK3, which were assessed by qRT-PCR analysis. Values are the mean with SD (n = 5). ∗*p* < 0.05; significant difference between the two groups (*t*_8_ = 2.682, *p* = 0.028; unpaired *t* test, two sided). RIP, RNA immunoprecipitation; TBP, TATA-binding protein.

In general, most circRNAs are produced by back-splicing, in which a downstream 5′ splice site is joined to an upstream 3′ splice site (15); thus, circRNAs and their counterpart mRNAs are produced from the same transcripts. Back-splicing is often induced by forming a double-dsRNA structure of inverted complementary sequences in introns flanking the circularized exon, which brings these splice sites into close proximity (45). CircHIPK3 is produced from around exon 2 of the *HIPK3* gene (46). Two inverted *Alu* elements were located in the flanking introns of exon 2 of the *HIPK3* gene (Fig. 6*A*). The *in silico* prediction analysis for the secondary structure of pre-mRNA of *HIPK3* gene also indicated that introns flanking exon 2 form a stable dsRNA of approximately 270 bp in length (Fig. 6*B*). The formed dsRNA structure was sufficiently long to be bound to and edited by ADAR1, which converts adenosines in long regions of dsRNA to inosines (25, 26). The RNA immunoprecipitation (RIP) analysis using exogenous RNA extracted from RPTECs infected with circHIPK3-expressing lentivirus revealed that ADAR1 bound to the dsRNA region in the precursor of circHIPK3 (*p* < 0.01, Fig. 6*C*), suggesting that the biogenesis of circHIPK3 is regulated by A-to-I RNA editing catalyzed by ADAR1 or its binding capacity.

The search for RNA editing sites using REDIportal, a comprehensive database (47), revealed that a total of 89 A-to-I RNA editing sites were registered in the flanking introns of *HIPK3* exon 2 (Fig. S4*A*). Introduction of mutations into these registered editing sites diminished the complementarity of the RNA pairs in precursor of circHIPK3, demonstrating that A-to-I RNA editing disrupts the RNA pairing (Fig. S4*B*). Direct sequencing analysis of exogenous RNA extracted from ADAR1-overexpressing or ADAR1-KD RPTECs infected with circHIPK3-expressing lentivirus also revealed three A-to-I RNA editing sites in the precursor of circHIPK3 (Figs. 6*D* and S4*A*). To explore whether ADAR1-mediated A-to-I RNA editing affects the biogenesis of circHIPK3, we constructed mutated circHIPK3 expression vectors in which the three adenosine editing sites were converted to guanine. The mutation destabilized the dsRNA structure in the precursor of circHIPK3 (Fig. 6*E*) and significantly reduced the biogenesis of circHIPK3 (*p* < 0.05, Fig. 6*F*). This suggested that ADAR1-mediated reduction of circHIPK3 levels was, at least in part, due to the RNA editing in the precursor of circHIPK3. This ADAR1-induced prevention of circHIPK3 biogenesis may enable miR-381-3p to interfere with translation from *ABCC4* mRNA to MRP4 protein.

## Discussion

**Figure 7:**
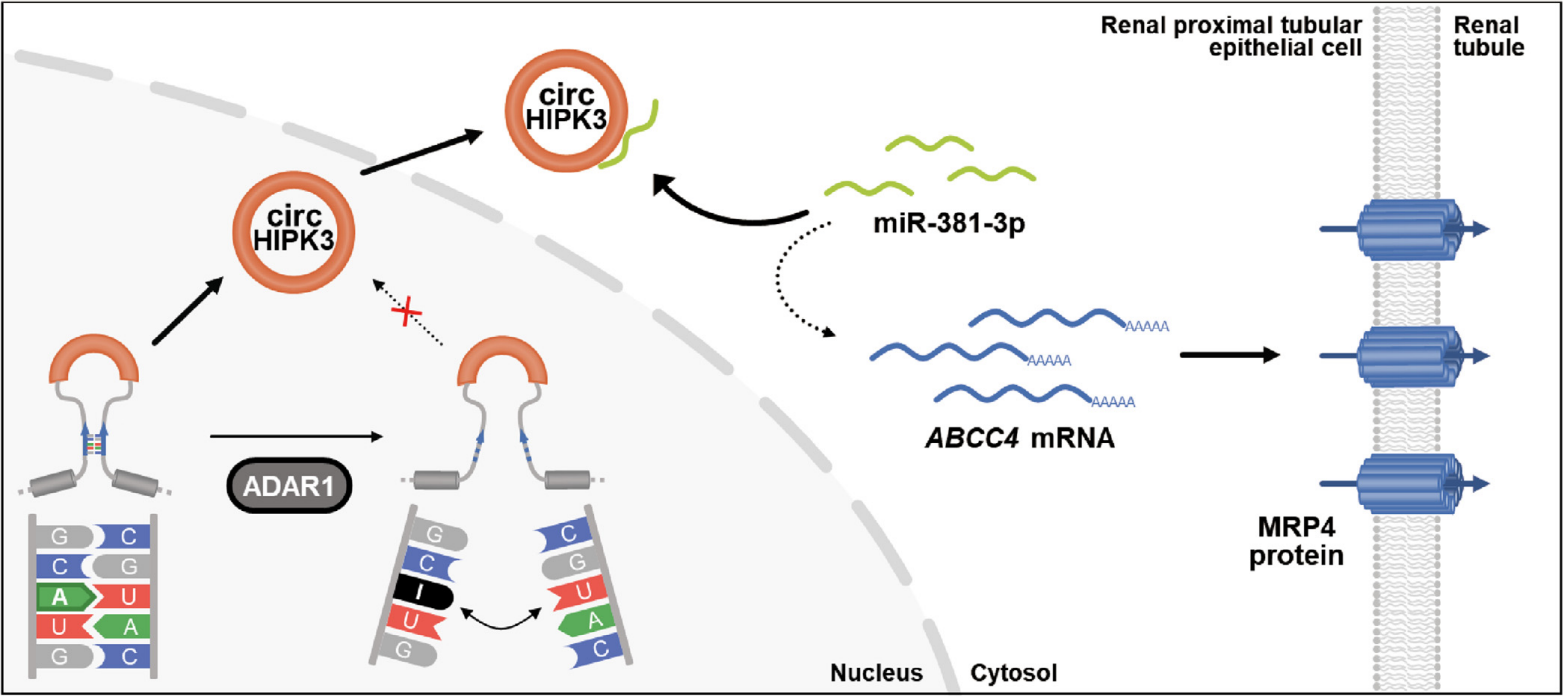
**Schematic diagram of the ADAR1-mediated regulation of MRP4 expression in human renal cells.** ADAR1 edits and destabilizes the dsRNA structure in the precursor of circHIPK3, thereby preventing circHIPK3 biogenesis. circHIPK3 binds to miR-381-3p and reduces its expression levels, promoting the translation from *ABCC4* mRNA to MRP4 protein. Thus, ADAR1-induced prevention of circHIPK3 biogenesis leads to the reduction of MRP4 expression *via* miR-381-3p.

MRP4 is an ABC transporter and is distributed in many tissues and cancer cells. As a xenobiotic transporter, MRP4 recognizes a variety of compounds as substrates and transports them to the extracellular fluid. The expression of MRP4 is regulated in a tissue-dependent manner through multiple processes, transcriptional regulations, posttranscriptional regulations, and membrane internalization (5, 48, 49). In this study, we demonstrated that the expression of MRP4 in human renal cells is regulated by the RNA editing enzyme ADAR1. A-to-I RNA editing catalyzed by ADAR1 reduced the production of circHIPK3, resulting in the increased expression of miR-381-3p, which suppressed the translation from *ABCC4* mRNA to MRP4 protein (Fig. 7).

Recent studies reported that several ABC transporters are subject to miRNA-mediated gene regulation (8). For example, *ABCC4* was reported to be regulated by miR-125a and miR-125b in hepatocellular carcinoma (6). The expression of miR-125a and miR-125b was also detected in RPTECs, but their expression levels were not affected by the downregulation of ADAR1. In addition, miR-124a and miR-506 were reported to downregulate MRP4 protein levels in HEK293T/17 cells (10). The expression levels of miR-124a and miR-506 were negatively correlated with MRP4 levels in the human kidney (10). Although the target site of miR-506, but not that of miR-124, was located in the 3′UTR of *ABCC4* mRNA, no significant expression of the miRNA was detected in RPTECs. By combining computational analysis using TargetScanHuman and molecular experiments, we identified miR-381-3p as a novel regulator of MRP4 expression. miR-381-3p has been reported to function in the regulation of cancer cell proliferation (50), inflammation response (51), and drug resistance (52). Similar to its effects on *ABCC4* mRNA, miR-381-3p targets the 3′UTR of *ABCB1* mRNA and promotes cisplatin sensitivity in breast cancer cells (52) This suggests that miR-381-3p is involved in the posttranscriptional regulation of other xenobiotic transporters. Further studies are required to investigate the effects of miR-381-3p on the regulation of MRP4 in other organs and under pathological conditions.

Recent evidence has demonstrated significant roles of circRNAs in the regulation of the immune system (53), cell proliferation (46), and neuronal function (54). CircRNAs have a wide range of functions in the regulation of gene expression. As the most elucidated function to date, circRNAs function as competing endogenous RNAs, which are defined as the sponge that entraps miRNAs and prevents their binding to target mRNAs (55). CircHIPK3 is one of the typical circRNAs acting as a competing endogenous RNA. For example, circHIPK3 was reported to promote cell proliferation by inhibiting the function of miR-124 (46). CircHIPK3-mediated regulation of miR-338-3p function also underlies the alteration of insulin secretion (56). In human hepatocellular carcinoma, circHIPK3 is suggested to regulate the expression of MRP4 *via* sponging miR-124-3p and miR-4524 (57). On the other hand, our study revealed that circHIPK3 regulates the expression of MRP4 *via* sponging miR-381-3p in human renal cells. This difference was probably due to miRNA expression profile, which varies in a tissue- and cell type–dependent manner (58). Indeed, the expression of miR-124-3p and miR-4524 was undetectable in RPTECs, and the expression level of miR-381-3p in the kidney was approximately 2.6-fold higher than that in the liver according to the human miRNA tissue atlas, a comprehensive database of miRNA expression (59). Consequently, the target and function of circRNAs depend on the expression levels of miRNA in each tissue or cell type.

Although circRNA entraps miRNAs thereby preventing their binding to target mRNAs, it remains to be clarified how circRNA decreases the expression levels of target miRNAs (60, 61). In this study, we demonstrated that the increased expression of circHIPK3 in RPTECs led to the destabilization of miR-381-3p and thus decreased its expression levels. In both intracellular and extracellular fluids, most miRNAs are thought to be stable. The degradation mechanism of miRNA, especially after being entrapped by circRNA, is yet to be elucidated (62). Further studies are required to uncover the circRNA-mediated regulation of miRNA stability and its degradation.

A-to-I RNA editing is the most prevalent nucleotide conversion in mammals (23). ADAR1 catalyzes A-to-I RNA editing on dsRNA in not only protein coding regions of mRNA, but also in noncoding regions that often contain *Alu* elements. Thus, ADAR1 has been thought to act as a regulator of miRNA production (63, 64). In this decade, circRNA was demonstrated to function as an miRNA sponge and also recognized as a promising novel noncoding RNA in gene regulation (12, 13). The biogenesis of circRNA is regulated by *cis*-regulatory elements and *trans*-acting factors. RNA pairing formed across the introns that flank the circularized exon is the most effective *cis*-regulatory element. ADAR1 acts as a *trans*-acting factor by disrupting the RNA pairing, resulting in the decreased formation of circRNA. The RNA editing enzyme converts adenosines to inosines in the dsRNA that is longer than approximately 50 bp (25, 26), which are often observed in the *cis*-regulatory elements of circRNA formation (65). Therefore, ADAR1 appears to exert a broad range of effects on circRNA expression. However, our understanding of the role of ADAR1 in the formation of circRNA is limited. Only a few studies have reported ADAR1-mediated circRNA expression (27, 28, 29). In the present study, we found that ADAR1 is involved in the regulation of circHIPK3 production.

Inosine is an analog of guanosine, forming base pairs with cytidine in a Watson–Crick bonding configuration. Thus, inosine is recognized as guanosine in downstream processes such as splicing and translation (23). Using these characteristics, A-to-I RNA editing is usually detected by Sanger sequencing; edited adenosine is read as guanosine in the electropherogram. We also used Sanger sequencing to detect and quantify A-to-I RNA editing in the precursor of circHIPK3. Three adenosines were considered to be edited because their editing levels increased and decreased in ADAR1-OX and in ADAR1-KD RPTECs, respectively. The editing levels of three adenosines were relatively low (less than 10%), which was possibly due to an experimental limitation. As the endogenous precursor of circHIPK3 was difficult to be detected even by PCR, cells were transfected with circHIPK3 expression vectors and their RNA was applied to Sanger sequencing analysis. The excessive abundance of circHIPK3 precursor, as an ADAR1 substrate, may lead to the underestimation of RNA editing levels. Furthermore, ADAR1 has been reported to function not only as an RNA editing enzyme but also as a dsRNA-binding protein (40, 41). The wildtype ADAR1 decreased the expression of MRP4 protein in RPTECs accompanying with decreasing and increasing the levels of circHIPK3 and miR-381-3p, respectively. However, the modulatory effects of ADAR1 on the expression of MRP4, circHIPK3, and miR-381-3p disappeared by mutating not only the catalytic deaminase domain but also the dsRNA-binding domains. Considering the multiple roles of ADAR1, its binding capacity to dsRNA may also play a role in the prevention of circHIPK3 biogenesis. Further studies are required to clarify the detailed role of ADAR1 in the regulation of circHIPK3 formation.

In this study, we demonstrated that ADAR1 regulates the expression of MRP4 *via* the circHIPK3/miR-381-3p regulatory network in human renal cells (Fig. 7). ADAR1 edits and destabilizes the dsRNA structure in the precursor of circHIPK3, thereby preventing its formation, whereas circHIPK3 exerts its sponging function against miR-381-3p, thereby preventing the miR-381-3p-mediated repression of translation from *ABCC4* mRNA to MRP4 protein. Consequently, ADAR1-induced prevention of circHIPK3 biogenesis may enable miR-381-3p to interfere with the expression of MRP4 protein. Although several regulatory factors have been identified to be involved in the expression process of MRP4 (5), we revealed that miR-381-3p acts as a novel regulator of MRP4 expression in human renal cells. In addition to MRP4, several ABC transporters are responsible for drug excretion. Hereafter, we should systematize the regulation mechanism underlying the function and expression of xenobiotic transporters and make a strategy to predict the excretion of drugs.

## Experimental procedures

### Cell culture and treatment

Human RPTECs (SA7K clone) were commercially obtained from Sigma-Aldrich. Cells were cultured under 5% CO_2_ at 37 °C in MEMα supplemented with 5.5% RPTEC Complete Supplement (Sigma-Aldrich), 2.33 mM l-glutamine (Sigma-Aldrich), 28 μM gentamicin (Sigma-Aldrich), and 14 nM amphotericin B (Sigma-Aldrich). For RNA stability assay, RPTECs were treated with 5 μM actinomycin D (FUJIFILM Wako Pure Chemical Corporation), and cells were collected for RNA extraction at the indicated time points. For RNase R treatment, 20 μg of total RNA was incubated at 37 °C for 20 min with or without 2 U/μg of RNase R (Applied Biological Materials Inc), and the RNA was then purified by the phenol–chloroform precipitation method.

### Construction of ADAR1-KD RPTECs

RPTECs were infected with lentiviral particles expressing small hairpin RNA (shRNA) against the human *ADAR1* gene (sc-37657-V; Santa Cruz Biotechnology), which contained three target-specific constructs encoding 19 to 25-nucleotide shRNA designed to repress the expression of ADAR1. To select clones stably expressing shRNA, cells were maintained in a medium containing 5 μg/ml of puromycin (FUJIFILM Wako Pure Chemical Corporation). Downregulation of ADAR1 was confirmed by Western blotting. To construct mock-transduced RPTECs as control cells, naive RPTECs were infected with lentivirus particles derived from pLVSIN-CMV Pur vector (Takara Bio Inc) and were cultured in puromycin-containing medium as described above to select the stably expressing cells.

### Western blotting

Total protein of RPTECs was prepared using CelLytic MT (Sigma-Aldrich) according to the manufacturer’s instructions. Denatured samples containing 5 μg of protein were separated by sodium dodecyl sulfate-polyacrylamide gel electrophoresis (SDS-PAGE) and then transferred onto polyvinylidene difluoride membranes. The membranes were incubated with primary antibodies against ADAR1 (1:1000, sc-73408; Santa Cruz Biotechnology), MRP4 (1:3000, ab15602; abcam), TBP (1:1000, ab51841; abcam), and β-ACTIN (1:10,000, sc-1616; Santa Cruz Biotechnology). Specific antigen–antibody complexes were visualized using horseradish peroxidase–conjugated anti-mouse antibodies (1:10,000, sc-2005; Santa Cruz Biotechnology) against ADAR1 and TBP, or anti-rat antibodies (1:10,000, ab97057; abcam) against MRP4 and ImmunoStar LD (FUJIFILM Wako Pure Chemical Corporation). Visualized images were scanned using ImageQuant LAS4010 (FUJIFILM).

### Quantitative and qualitative RT-PCR analysis for mRNA and circRNA

Total RNA was extracted from RPTECs using RNAiso Plus (Takara Bio Inc) according to the manufacturer’s instructions. The extracted RNA was reversed transcribed using ReverTra Ace qPCR RT Kit (TOYOBO Co, Ltd). For quantitative real-time RT-PCR, the cDNA equivalent of 10 ng of RNA was amplified by PCR using the LightCycler 96 system (Roche Diagnostics) with THUNDERBIRD Next SYBR qPCR Mix (TOYOBO Co, Ltd). For qualitative RT-PCR, the cDNA equivalent of 10 ng of RNA was amplified by the GoTaq Green Master Mix (Promega) with the divergent or convergent primers listed in Tables 1 and S1. The PCR-amplified products were separated by electrophoresis using 2% agarose gel containing ethidium bromide. Signals from the agarose gel were detected using LAS3000 (FUJIFILM). PCR-amplified products were confirmed by Sanger sequencing.

**Table 1:** Primer sets for quantitative and qualitative RT-PCR analysis of gene expression

Gene	Primers
Human *ABCC4*	
Forward	5′-AGTCAATTCTGAAAGCTCCGGTA-3′
Reverse	5′-CGGCAGCAAATCATCCAAGTG-3′
Human *HIPK3*	
Forward	5′-GCACATGTTGTCTGGCCTCAG-3′
Reverse	5′-TCTCTTCCTGGCTCCCATTCC-3′
Human circHIPK3 [divergent]	
Forward	5′-TATGTTGGTGGATCCTGTTCGGCA-3′
Reverse	5′-TGGTGGGTAGACCAAGACTTGTGA-3′
Human circHIPK3 [convergent]	
Forward	5′-TTAGTCCCCTGCCACTAAAAGTGA-3′
Reverse	5′-TGCCGAACAGGATCCACCAACATA-3′
Human *β-ACTIN* [divergent]	
Forward	5′-CACCTTCACCGTTCCAGTTT-3′
Reverse	5′-AGTCATTCCAAATATGAGATGCG-3′
Human *β-ACTIN* [convergent]	
Forward	5′-AAACTGGAACGGTGAAGGTG-3′
Reverse	5′-CGCATCTCATATTTGGAATGACT-3′
Human pre-circHIPK3	
Forward	5′-TATTTGCACTGCCAACTAGTTAAG-3′
Reverse (qRT-PCR)	5′-CTGAATCCTTATACAGGTTGAGCA-3′
Reverse (direct sequencing)	5′-CTAAAGCGCATGCTCCAGAC-3′
Human 18S rRNA	
Forward	5′-AGAAGCCCCTGGCACTCTAT-3′
Reverse	5′-GCAAAGTGGGCACAGTGATG-3′
*Luciferase*	
Forward	5′-CAGCGAGAATAGCTTGCAGT-3′
Reverse	5′-AGGATCTTTTGCAGCCCTTT-3′
*Renilla Luciferase*	
Forward	5′-GAGAAGGGCGAGGTTAGACG-3′
Reverse	5′-CCCGAAGGTAGGCGTTGTAG-3′

### Quantitative RT-PCR analysis for miRNA

Total RNA was extracted from RPTECs using the miRNeasy Mini Kit (QIAGEN) according to the manufacturer’s instructions. The extracted RNA was reverse transcribed using the Taqman Advanced miRNA cDNA Synthesis Kit (Applied Biosystems). Quantitative real-time PCR analysis was performed using the 7500 Real-time PCR system with Taqman Fast Advanced Master Mix and miRNA-specific Taqman Advanced MicroRNA Assays for miR-381-3p (477816_mir; Applied Biosystems) and miR-191-5p (477952_mir; Applied Biosystems). The expression levels of miR-381-3p were normalized to those of miR-191-5p, which has been recommended as an endogenous control of miRNA (32).

### Plasmid construction

The upstream region (from bp −1650 to +70, where +1 indicates the transcription start site) and 3′UTR (from bp +1 to +1741, to +1192 and to +553, where +1 indicates the stop codon) of the human *ABCC4* gene were amplified using PrimeSTAR MAX DNA polymerase (Takara Bio Inc). These sequences were subcloned into the pGL4.25 luciferase-reporter vector (Promega) using KpnI and NheI restriction sites and into the pGL4.13 luciferase-reporter vector (Promega) using XbaI and FseI restriction sites. To construct the reporter vector with mutation in miR-381-3p target site, the complementary sequence of the seed region of miR-381-3p (5′-TTGTATA-3′) on *ABCC4* 3′UTR::Luc was mutated to the MluI restriction site (5′-ACGCGTA-3′) using the PrimeSTAR Mutagenesis Basal Kit (Takara Bio Inc) according to the manufacturer’s protocol.

The expression vector of human ADAR1 with C-terminal GFP-tag was commercially obtained from Origene Technologies, Inc (RG219761). To construct the RNA editing activity–deficient ADAR1 (E617A-mutated ADAR1) expression vector, the DNA sequence of Glu617 (GAA) was mutated to Ala617 (GCA). To construct the dsRNA binding ability–deficient ADAR1 (EAA-mutated ADAR1) expression vector, each DNA sequence of KKxxK motif in the dsRBD was mutated to EAxxA motif (dsRBD#1: 5′-AAGAAAGTGGCCAAG-3′ to 5′-GAGGCCGTGGCGGCG-3′; dsRBD#2: 5′-AAGAAAGTGGCAAAG-3′ to 5′-GAGGCAGTGGCAGCG-3′; dsRBD#3: 5′-AAGAAGCAAGGCAAG-3′ to 5′-GAGGCCCAAGGGGCG-3′), using the PrimeSTAR Mutagenesis Basal Kit (Takara Bio Inc) according to the manufacturer’s protocol.

The precursor sequence of circHIPK3 was amplified using PrimeSTAR MAX DNA polymerase (Takara Bio Inc). The sequence was subcloned into the pcDNA3.1 vector (Invitrogen) using NheI and KpnI restriction sites and into the pLVSIN-CMV Pur vector (Takara Bio Inc) using XbaI and NotI restriction sites. To construct the circHIPK3 expression vector with edited-form mutation in the *Alu* element, the unedited sequence (5′-CTAAAAATA-3′) in the circHIPK3 expression vector was mutated to the edited sequence (5′-CTAGGGATA-3′) using the PrimeSTAR Mutagenesis Basal Kit (Takara Bio Inc) according to the manufacturer’s protocol. Sequences of primers are listed in Table 2.

**Table 2:** Primer sets for plasmid construction

Gene	Primers
Human *ABCC4* upstream	
Forward for −1651 bp	5′-CGTGGTACCCAGGGTGGATATGAAGAGCAGC-3′
Reverse for +70 bp	5′-ATAGCTAGCCCTGGACCTCAAGCAGGGATG-3′
Human *ABCC4* 3′UTR	
Forward for +1 bp	5′-GCGCTCTAGAATCCAACCAAAATGTCAAGTC-3′
Reverse for +1741 bp	5′-TAGGCCGGCCATGGAGATGAAAACTATCATTTA-3′
Reverse for +1192 bp	5′-TAGGCCGGCCACTCAACATATTACAGCCAC-3′
Reverse for +553 bp	5′-TAGGCCGGCCGGTACACACTCCCTACTA-3′
Human *ABCC4* 3′UTR mutation	
Forward	5′-TTGTACGCGTACATATATTTGTCCTTCG-3′
Reverse	5′-TGTACGCGTACAAATGCACACGTG-3′
Human circHIPK3 expression into pcDNA3.1	
Forward	5′-CGTGCTAGCTGAGGCAATTCTTGGGCTTTAGG-3′
Reverse	5′-CGCGGTACCAGTCACGGGAGCCAAAGAAGTAT-3′
Human circHIPK3 expression into pLVSIN-CMV Pur	
Forward	5′-CGGCTCTAGATGAGGCAATTCTTGGGCTTTAGG-3′
Reverse	5′-ATTGCGGCCGCAGTCACGGGAGCCAAAGAAGTAT-3′
Human circHIPK3 expression mutation	
Forward	5′-CTCTACTAGGGATAACAAAAATTAGCTGG-3′
Reverse	5′-TGTTATCCCTAGTAGAGATGGGGGTTT-3′
Human ADAR1 E617A-mut	
Forward	5′-CATGCAGCAATAATCTCCCGGAGAG-3′
Reverse	5′-GATTATTGCTGCATGGCAGTCATTGAC-3′
Human ADAR1 EAA-mut#1	
Forward	5′-GAGGCCGTGGCGGCGCAGGATGCAGCTATGA-3′
Reverse	5′-CGCCGCCACGGCCTCGCTTCCAGCTTCAG-3′
Human ADAR1 EAA-mut#2	
Forward	5′-GAGGCAGTGGCAGCGCAGATGGCCGCAGA-3′
Reverse	5′-CGCTGCCACTGCCTCGCTGGGAGCACTCAC-3′
Human ADAR1 EAA-mut#3	
Forward	5′-GAGGCCCAAGGGGCGCAGGAAGCAGCAGATG-3′
Reverse	5′-CGCCCCTTGGGCCTCGCTGTGTGCGCAG-3′

The numbers indicate the distance from the transcription site (+1) for the upstream vector or from the stop codon (+1) for the 3′UTR vector.

### Luciferase reporter assay

RPTECs were seeded at a density of 2.0 × 10^5^/well in 24-well culture plates. Lipofectamine LTX regent (Invitrogen) was used for reverse transfection with 500 ng of the pGL4.25 reporter construct or 200 ng of the pGL4.13 reporter constructs. Ten nanograms of the pRL-TK vector (Promega) was also transfected as an internal control reporter. Cells were harvested 24 h after transfection, and lysates were analyzed using the Dual-Luciferase reporter assay system (Promega) with Berthold Lumat3 LB9508 (Berthold Japan Co, Ltd). The ratio of firefly (derived from pGL4.25 and pGL4.13 reporter constructs) to Renilla (derived from pRL-TK) luciferase activity in each sample served as a measure of normalized luciferase activity.

### miRNA microarray analysis

Total RNA was extracted from mock-transduced and ADAR1-KD RPTECs using the miRNeasy Mini Kit (QIAGEN). The quality of the RNA was checked with Experion (Bio-Rad). In total, 2549 miRNAs were scanned on the SurePrint miRNA Microarray rel. 21 (Agilent Technologies). To identify upregulated or downregulated miRNAs, we calculated ratios and Z-scores from the normalized signal intensities of each probe. Two criteria were set as follows: for upregulated miRNAs, a ratio of 2.0-fold or Z-score of 2.0; for downregulated miRNAs, a ratio of 0.5-fold or Z-score of -2.0. The full data have been deposited in National Center for Biotechnology Information gene expression omnibus (Accession#: GSE192692).

### Overexpression of ADAR1 with electroporation

The suspension of RPTECs (1.0 × 10^6^ cells) was mixed with 10 μg of ADAR1-expression plasmids or pcDNA3.1 in Opti-MEM I Reduced Serum Medium (Thermo Fisher Scientific). The cells–plasmids suspension was then transferred to a cuvette, and the plasmids were transferred to the cells by electroporation using Super Electroporator NEPA21 (NEPA GENE, Co, Ltd). Poring pulses were applied at 175 V (pulse length, 5 ms; pulse interval, 50 ms; number of pulses, 2; decay rate, 10%), followed by transfer pulses at 20 V (pulse length, 50 ms; pulse interval, 50 ms; number of pulses, 5; decay rate, 40%).

### *In silico* prediction of RNA secondary structure

The secondary structure of the human *HIPK3* pre-mRNA was predicted using RNAfold (66), which computes the minimum free energy and predicts an optional secondary structure (http://rna.tbi.univie.ac.at/cgi-bin/RNAWebSuite/RNAfold.cgi). The range from −630 to +2060 of *HIPK3* exon 2 was input as data with all default options.

### RNA immunoprecipitation

RPTECs were infected with lentiviral particles expressing precursor of circHIPK3. The lentivirus particles were prepared by the Lentiviral High Titer Packaging Mix with Lenti-X 293T Cell Line (Takara Bio Inc). Forty-eight hours after transduction, cells were collected and lysed in 1 ml of nuclear isolation buffer (1.28 M sucrose, 40 mM Tris-HCl pH 7.5, 20 mM MgCl_2_, and 4% Triton X-100). After the addition of 1 ml of PBS and 3 ml of distilled water to lysates, they were centrifuged at 2500*g* for 15 min at 4 °C, and resuspended in RIP buffer (150 mM KCl, 25 mM Tris-HCl pH 7.4, 5 mM EDTA, 0.5 mM dithiothreitol, 0.5% Nonidet P-40, 2 μg/ml of leupeptin, 2 μg/ml of aprotinin, and 100 U/ml of recombinant RNase inhibitor (TOYOBO Co, Ltd). The resuspended nuclei were homogenized and centrifuged at 13,000 rpm for 10 min, and the supernatant was then incubated with anti-ADAR1 antibody (sc-73408; Santa Cruz Biotechnology) or mouse IgG (Fujifilm Wako Pure Chemical) for 2 h at 4 °C with gentle rotation, followed by incubation with protein G beads (Thermo Fisher Scientific) for 1 h at 4 °C. Then, the beads were washed with RIP buffer three times. A total of 10% of beads was used for protein elution, while the rest was subjected to RNA extraction using QIAzol Lysis Reagent and miRNeasy Mini Kit (QIAGEN) and used for quantitative RT-PCR (qRT-PCR) analysis as mentioned above. During RNA extraction from immunoprecipitants, DNase treatment was conducted to avoid contamination of the genomic DNA. %Input = 2^−ΔCt^ × 100: ΔCt = Ct_RIP_ − [Ct_input_ − Log_2_(input dilution factor)]. Sequences of primers for amplification of the precursor of circHIPK3 (pre-circHIPK3) are listed in Table 1.

### Direct sequencing

RPTECs were infected with lentiviral particles expressing the precursor of circHIPK3. RNA was extracted using the ReliaPrep RNA Cell Miniprep system (Promega) and treated with DNase I on columns. A total of 500 ng of RNA was used for cDNA synthesis with ReverTra Ace qPCR RT Kit (TOYOBO Co, Ltd). DNA was extracted using the Wizard Genomic DNA Purification Kit (Promega). The cDNA and DNA were amplified by the GoTaq Green Master Mix (Promega) with primers listed in Table 1. The PCR-amplified products were analyzed by Sanger sequencing using the same forward primer used in the PCR (Human pre-circHIPK3 in Table 1). Electropherograms were aligned using SnapGene Viewer (https://www.snapgene.com/snapgene-viewer/).

### Statistical analysis

All statistical analyses were carried out using JMP pro 14 (SAS institute Japan). The significance of differences among groups was analyzed by ANOVA, followed by Tukey–Kramer’s and Dunnett’s post hoc test. The unpaired *t* test was used for the comparison of data between two groups. Equal variances were not formally tested. A 5% level of probability was considered to be significant.

## Data availability

All data supporting the results of the present study are included in the article. The full data of microarray analysis were deposited in National Center for Biotechnology Information gene expression omnibus (Accession#: GSE192692).

## Supporting information

This article contains supporting information.

## Conflict of interest

The authors declare that they have no conflicts of interest with the contents of this article.
