# Supplementary material for: RNA editing enzyme ADAR1 controls miR-381-3p-mediated expression of multidrug resistance protein MRP4 via regulation of circRNA in human renal cells
Source: J Biol Chem. 2022 Jun 23;298(8):102184. doi: 10.1016/j.jbc.2022.102184 (PMC9293778; doi:10.1016/j.jbc.2022.102184)
Supplement: Supplemental Table S1 and Figures S1–S4 [file mmc1.pdf]

RNA editing enzyme ADAR1 controls miR-381-3p-mediated expression of multidrug resistance protein MRP4 via regulation of circRNA in human renal cells

Yuji Omata, Maseri Okawa, Mai Haraguchi, Akito Tsuruta,
Naoya Matsunaga, Satoru Koyanagi, Shigehiro Ohdo

Supporting Information

EXPERIMENTAL PROCEDURES

Supplementary Table S1
Primer sets for quantitative RT-PCR analysis for circRNA expression.

Supplementary Figure S1
Effects of the knockdown of ADAR1 on the transcription activity of the *ABCC4* gene.

Supplementary Figure S2
Electropherograms from direct sequencing of the PCR-amplified product of *ABCC4* 3′ UTR.

Supplementary Figure S3
Effect of the knockdown of ADAR1 on the expression levels of circRNAs highly expressed in RPTECs.

Supplementary Figure S4
Estimation the effects of ADAR1-mediated A-to-I RNA editing on the secondary structure of the precursor of circHIPK3.

EXPERIMENTAL PROCEDURES

Direct sequencing

RNA was extracted from RPTECs using the ReliaPrep RNA Cell Miniprep system (Promega) and treated with DNase I on columns. A total of 500 ng of RNA was used for cDNA synthesis with ReverTra Ace qPCR RT Kit (TOYOBO Co., Ltd.). Genomic DNA was extracted using the Wizard Genomic DNA Purification Kit (Promega). The cDNA and genomic DNA were amplified by the GoTaq Green Master Mix (Promega) with the same gene-specific primers used in plasmid construction (Human *ABCC4* 3′UTR; **Table 2**). The PCR-amplified products were analyzed by Sanger sequencing using the same primer used in the PCR (*ABCC4* 3′UTR Reverse for +1741 bp; **Table 2**). Electropherograms were aligned using SnapGene Viewer.

Table S1 Primer sets for quantitative RT-PCR analysis of gene expression

circRNA		Primers
hsa_ZNF124_0005		
	Forward	5′-TAAGCAATAAGAACTCGGTTGCC-3′
	Reverse	5′-GGGAAGGATCCAACAAAGCCC-3′
hsa-RBM33_0009		
	Forward	5′-CCCAGAAGAAGGACAGTATGAA-3′
	Reverse	5′-TGTAACACCCTGAGAACTGAAAT-3′
hsa-CGNL1_0010		
	Forward	5′-GGGAGCCCTGAAAGAAGAGGT-3′
	Reverse	5′-TGGAATTACAAGCCTTGGTTGCT-3′
hsa-ZKSCAN1_0001		
	Forward	5′-GAAACCCCGCCTCTTACA-3′
	Reverse	5′-TTCCTCTTCCACCTTCACTATTA-3′
hsa-CDYL_0005		
	Forward	5′-CTTAGCTGTTAACGGGAAA-3′
	Reverse	5′-CTGTTGAAGTCGTGGATGT-3′
hsa-MAN1A2_0003		
	Forward	5′-TCTGTGTTTGAAGTCAACATTCG-3′
	Reverse	5′-GCTTCTTCCAAGGCCTTCTC-3′
hsa-C3_0002		
	Forward	5′-CGGACAAGAAAGGTAACCTGGA-3′
	Reverse	5′-AAGTCCTCAACGTTCCACAGC-3′
hsa-FBXW7_0005		
	Forward	5′-AGTATTGTGGACCTGCCCGTTC-3′
	Reverse	5′-GCCAGCTTGCTACTTCTTGGA-3′
hsa-SLC8A1_0015		
	Forward	5′-ATCGAAGGGACTGCCAGAGG-3′
	Reverse	5′-GGTGAAAGACTTAATCGCCGC-3′
hsa-PCMTD1_0002		
	Forward	5′-CTGAAGTTATGGAAGCATTG-3′
	Reverse	5′-ATGGCTTCCAATATTGCACTTG-3′
hsa-PSD3_0018		
	Forward	5′-GTCCATTGCCTTACCTGTGCAA-3′
	Reverse	5′-TTGGCTGCTTCCACATTGCT-3′
hsa-SMARCA5_0005		
	Forward	5′-GAAAAGCTCTCCAAGATGGG-3′
	Reverse	5′-CTTCTTTGCACCTCTTTCCAA-3′
hsa-FGGY_0001		
	Forward	5′-TCTCCCGGACTTCTTATCGTGG-3′
	Reverse	5′-CGATGGTCCAGCCACATGAT-3′
hsa-RHOBTB3_0011		
	Forward	5′-AGGCAACCCACCATTACGAG-3′
	Reverse	5′-ATGGCTTACAGCGCAGAGAA-3′

hsa-FNDC3B_0004		
	Forward	5′-CAAGAAGCAGCCCAAAGTCG-3′
	Reverse	5′-CATGGCTGAGGGGTAGCTTG-3′
hsa-ATRNL1_0001		
	Forward	5′-CCAATTGCGGCAGTCCAGAT-3′
	Reverse	5′-TGGATAGCCTTCAATGAGCCA-3′
hsa-NOX4_0018		
	Forward	5′-TCCGGAGCAATAAGCCAGTC-3′
	Reverse	5′-TTAAGACTGATGCAGCCGGG-3′
hsa-SETD3_0001		
	Forward	5′-AAACACAGCTCGACAGTACGC-3′
	Reverse	5′-GCATTTCTGCAGCAGCTCAC-3′
hsa-RAB3IP_0013		
	Forward	5′-GTTCTGGAGGCTGTGGAAAA-3′
	Reverse	5′-GACACGTCCTGTCCATTGTG-3′
hsa-SLTM_0001		
	Forward	5′-AGAGGACATCGAAAGTCAGGAAA-3′
	Reverse	5′-TCCACAGAAGCATCTCCACTCA-3′
hsa-MAN1A2_0002		
	Forward	5′-GCAAGGAAAGGACACTCCCC-3′
	Reverse	5′-TCTTCCTCTTCCCACACTGAAA-3′
hsa-PTK2_0007		
	Forward	5′-TTGCCAAAAGGATTTCTAAACCAGT-3′
	Reverse	5′-GTTGGGGTCAAGGTAAGCAGC-3′
Human *GAPDH*		
	Forward	5′-ACAACTTTGGTATCGTGGAAGG-3′
	Reverse	5′-GCCATCACGCCACAGTTTC-3′

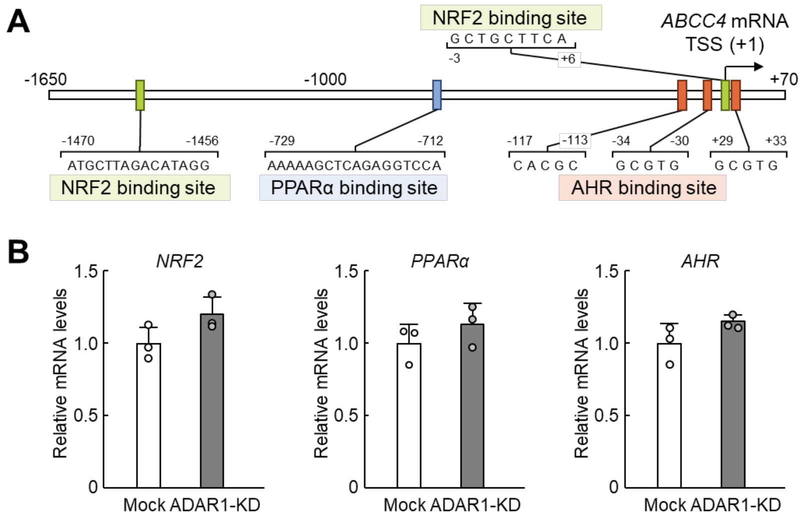

Supplementary Figure S1. Effects of the knockdown of ADAR1 on the transcription activity of the *ABCC4* gene. *A*, schematic diagrams of the 5'-flanking region of the *ABCC4* gene and response elements of well-known transcription factors. The numbers indicate the distance in base pairs from the putative transcription start site (TSS, +1). Binding sites of the transcription factors were searched in JASPAR2022 (https://jaspar.genereg.net/) and referred from a previous study (48). *B*, the mRNA levels of *NRF2*, *PPARα* and *AHR* in mock-transduced and ADAR1-KD RPTECs. The mRNA levels were normalized to those of 18S rRNA. Values are the mean with S.D. (n = 3).

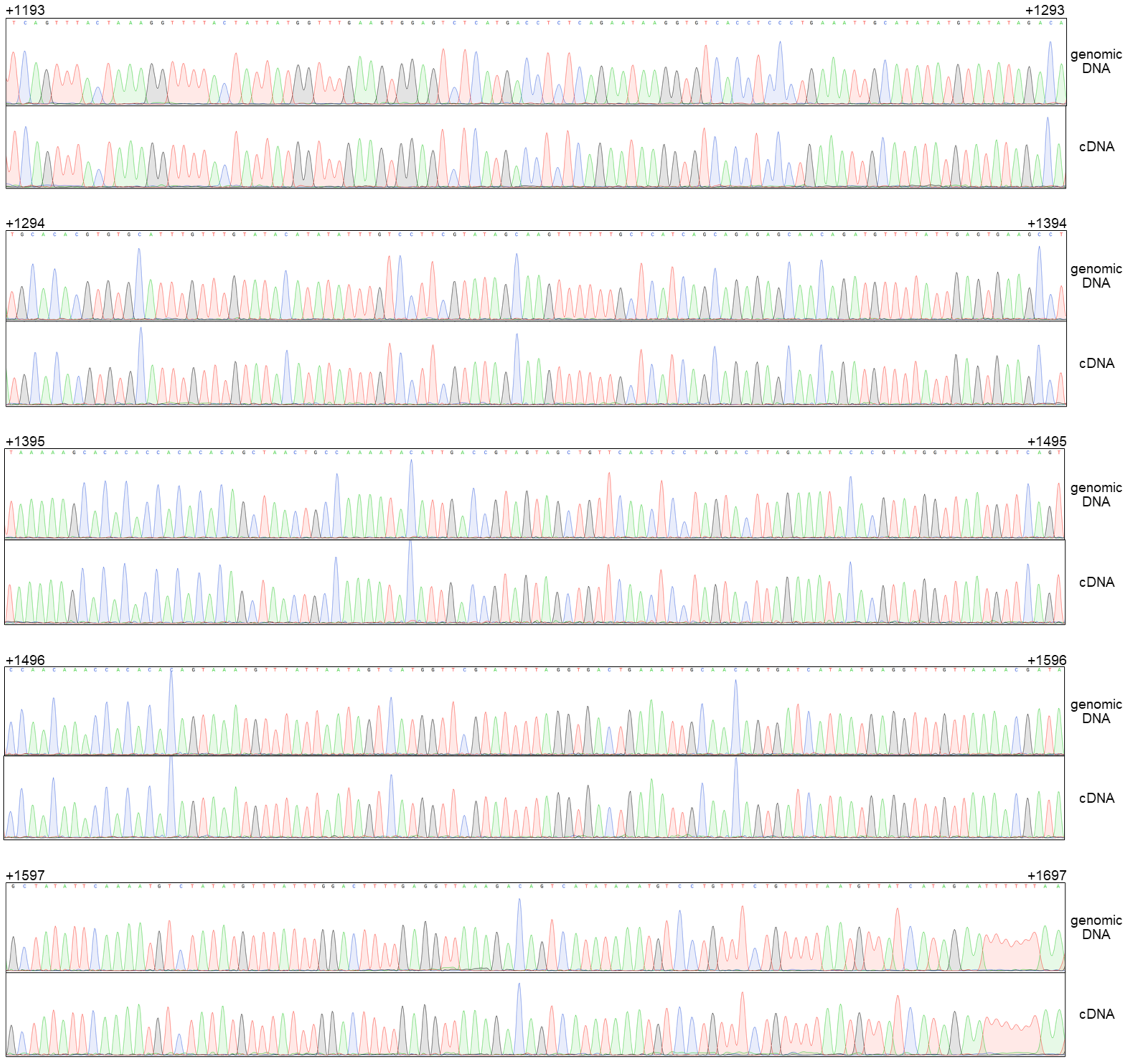

Supplementary Figure S2. Electropherograms from direct sequencing of the PCR-amplified product of *ABCC4* 3′UTR. The sequence data were obtained from genomic DNA and mRNA extracted from RPTECs. The numbers indicate the distance in base pairs from the stop codon (+1). Electropherograms were aligned using SnapGene Viewer.

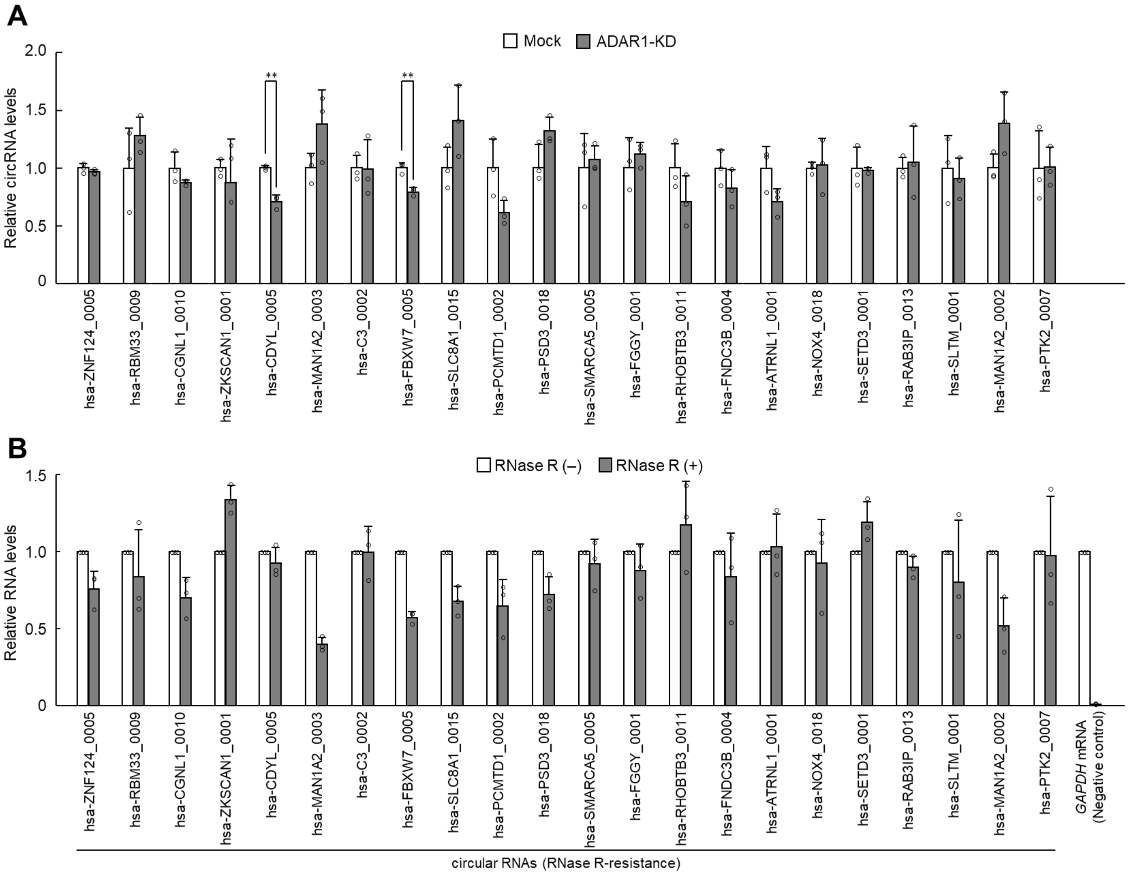

Supplementary Figure S3. Effect of the ADAR1 knockdown on the levels of circRNAs expressed in RPTECs. *A*, the expression levels of circRNAs listed in **Fig. 3A** in mock-transduced and ADAR1-KD RPTECs. The expression levels were normalized to those of 18S rRNA. Values are the mean with S.D. (n = 3). $^{**}p < 0.01$; significant difference between the two groups (t_4 = 8.067, p = 0.001 for circCDYL; t_4 = 6.503, p = 0.003 for circFBXW7; unpaired *t*-test, two-sided). *B*, qRT-PCR analysis for the abundance of circRNAs listed in **Fig. 3A** and *GAPDH* mRNA in RPTECs treated with RNase R. The amounts of target RNAs were normalized to those in the mock treatment.

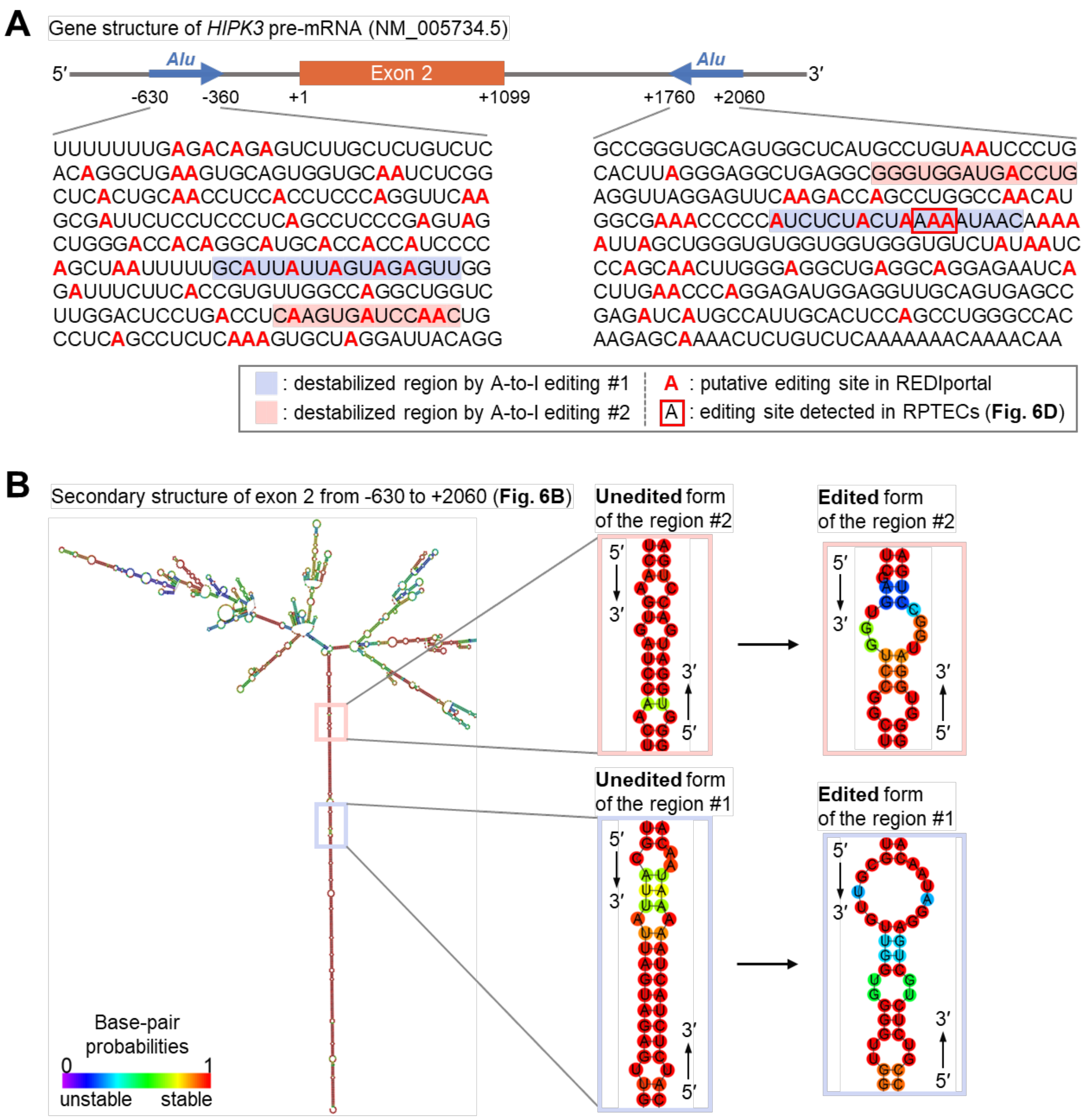

Supplementary Figure S4. Estimation the effects of ADAR1-mediated A-to-I RNA editing on the secondary structure of the precursor of circHIPK3. *A*, schematic diagram of the genomic region of human *HIPK3* exon 2 and its flanking introns with inverted *Alu* elements and detailed sequence of the inverted *Alu* elements. Sequences colored blue or red indicate the putative destabilized regions by A-to-I RNA editing. "A" colored red indicates A-to-I RNA editing sites registered in REDIportal. "A" enclosed in a red square indicates the editing sites detected in RPTECs with direct sequencing. *B*, *in silico* prediction of RNA secondary structure of the putative destabilized regions in precursor of circHIPK3. The *Middle panel* shows the unedited form and the *right panel* shows the edited form of the putative destabilized regions. The minimum free energy structure with base-pair probabilities was calculated to have the lowest value of free energy. Base-pair probabilities are shown by a color spectrum. In the l*eft panel,* the RNA secondary structure of the precursor of circHIPK3 shown in **Fig. 6B** was reused to show the location of the putative destabilized regions by A-to-I RNA editing.
